# Are hospital nurse staffing practices associated with postoperative cardiac events and death? A systematic review

**DOI:** 10.1371/journal.pone.0223979

**Published:** 2019-10-17

**Authors:** Jonathan Bourgon Labelle, Li-Anne Audet, Paul Farand, Christian M. Rochefort

**Affiliations:** 1 Division of Cardiology, Centre Hospitalier Universitaire de Sherbrooke, Sherbrooke, Quebec, Canada; 2 Centre de Recherche du Centre Hospitalier Universitaire de Sherbrooke, Sherbrooke, Quebec, Canada; 3 Centre de Recherche Charles-Le Moyne Saguenay-Lac-St-Jean sur les Innovations en Santé, Longueuil, Quebec, Canada; 4 School of Nursing, Faculty of Medicine and Health Sciences, Université de Sherbrooke, Sherbrooke, Quebec, Canada; Victoria University, AUSTRALIA

## Abstract

**Introduction:**

Postoperative cardiac events are frequent complications of surgery, and their occurrence could be associated with suboptimal nurse staffing practices, but the existing evidence remains scattered. We systematically reviewed studies linking nurse staffing practices to postoperative cardiac events and two related outcomes, all-cause mortality and failure-to-rescue.

**Methods:**

A systematic search of the English/French literature was undertaken in the CINAHL, PsychInfo, and Medline databases. Studies were included if they: a) were published between 1996 and 2018; b) used a quantitative design; c) examined the association between at least one of seven staffing practices of interest (i.e., staffing levels, skill mix, work environment characteristics, levels of education and experience of the registered nurses, and overtime or temporary staff use) and postoperative cardiac events, mortality or failure-to-rescue; and d) were conducted among surgical patients. Data extraction, analysis, and synthesis, along with study methodological quality appraisal, were performed by two authors. High methodological heterogeneity precluded a formal meta-analysis.

**Results:**

Among 3,375 retrieved articles, 44 studies were included (39 cross-sectional, 3 longitudinal, 1 case-control, 1 interrupted time series). Existing evidence shows that higher nurse staffing levels, a higher proportion of registered nurses with an education at the baccalaureate degree level, and more supportive work environments are related to lower rates of both 30-day mortality and failure-to-rescue. Other staffing practices were less often studied and showed inconsistent associations with mortality or failure-to-rescue. Similarly, few studies (n = 10) examined the associations between nurse staffing practices and postoperative cardiac events and showed inconsistent results.

**Conclusion:**

Higher nurse staffing levels, higher registered nurse education (baccalaureate degree level) and more supportive work environments were cross-sectionally associated with lower 30-day mortality and failure-to-rescue rates among surgical patients, but longitudinal studies are required to corroborate these associations. The existing evidence regarding postoperative cardiac events is limited, which warrants further investigation.

## Introduction

The World Health Organization estimates that between 266.2 and 359.5 million surgeries were performed in 2012 among its member states, an increase of 38% since 2005 [1]. During their recovery, a large portion of surgical patients are vulnerable to postoperative cardiac events (PCEs), which include myocardial infarction, dysrhythmias, and congestive heart failure [2]. Indeed, PCEs are estimated to develop within 30 days of a surgical procedure in more than 10 million adults worldwide each year, with incidence rates ranging between 1.0% and 7.0% depending on the surgical populations studied [2–4]. PCEs are currently the third leading cause of perioperative death in the United States [5] and are associated with serious morbidity [2,6–10]. Moreover, PCEs can increase the cost of hospitalization by up to 65% [11]. Given these figures, reducing the incidence of PCEs has been identified as a high priority worldwide [2,5].

To this end, researchers have identified several modifiable and nonmodifiable risk factors of PCE occurrence, including patient age and comorbidities, the type of surgical procedure performed, lifestyle habits, severity of illness, and the type of hospital admission (i.e., elective or urgent) [5,12]. Moreover, numerous interventions specifically targeting these risk factors have been proposed, such as the use of pharmacoprophylaxis (e.g., aspirin, beta blockers), systematic risk factor screening (e.g., Revised Cardiac Risk Index score), and the promotion of healthy lifestyles prior to a surgical procedure (e.g., smoking cessation, physical activity) [13]. However, none of these interventions, alone or in combination, has been fully effective at reducing PCE rates, and new risk factors and interventions must now be considered.

Among these, a growing number of studies have reported that several nurse staffing practices in hospitals (e.g., adequate staffing levels, a richer registered nurse (RN) skill mix, a higher proportion of RNs educated at the baccalaureate degree level, a greater work experience, more supportive work environments, and lower overtime and temporary staff use) are associated with lower rates of mortality and adverse events [14–16]. To explain these associations, it has been proposed that these staffing practices have the potential to enhance or weaken nurse surveillance, an important function of RNs in hospitals [17,18].

Specifically, nurse surveillance is the ongoing process through which RNs monitor patients for early signs of deterioration or complications in care and subsequently implement interventions that are required to minimize their impact on patient health and outcomes [17,19–23]. Adequate staffing levels and a richer RN skill mix are expected to increase the effectiveness of nurse surveillance by augmenting each RN’s time for direct patient care and the rapidity with which they can detect any change in a patient’s condition [17,19]. Supportive work environments provide RNs with greater decision-making autonomy and flexibility, which increase the timeliness of interventions once a potential problem in care has been identified [17]. Conversely, higher usage of overtime has been associated with increased fatigue and reduced vigilance, which may lessen the effectiveness of RN surveillance [24]. Similarly, knowledge of and familiarity with a given nursing unit’s policies and procedures is essential for effective RN surveillance, which may be reduced when greater proportions of temporary staff are used [25,26]. Last, a higher proportion of RNs educated at the baccalaureate degree level and a greater work experience are assumed to improve nurse surveillance by providing RNs with more knowledge, better patient surveillance skills, and a broader repertoire of interventions [17].

While there is growing international evidence suggesting that these nurse staffing practices are linked to improved outcomes among various populations of hospitalized patients, the evidence regarding PCEs (or their ultimate outcomes, death and failure-to-rescue, i.e., death following potentially preventable hospital-acquired complications [27]) in surgical patients remains scattered [14–16], which preclude evidence-based staffing decisions in this setting. Therefore, we aim to contribute to this field by 1) systematically reviewing the evidence on the associations between seven common nurse staffing practices (i.e., nurse staffing levels, RN skill mix, work environment characteristics, RN levels of education and experience, and the usage of overtime hours or temporary staff) and the occurrence of PCEs, all-cause mortality, and failure-to-rescue in surgical settings and 2) identifying avenues for further research.

## Materials and methods

### Design

A systematic review of the literature was performed according to the Preferred Reporting Items for Systematic Reviews and Meta-Analyses (PRISMA) guidelines (S1 Table) [28]. No published protocol is available for this study.

### Search strategy and inclusion criteria

Two research team members (JBL and LAA), with the assistance of an experienced medical librarian, independently searched the literature using the Population, Intervention-Comparator, Outcome, and Time (PICOT) framework [29] to identify studies that examined the associations between nurse staffing practices and the occurrence of PCEs, all-cause mortality or failure-to-rescue among surgical patients. Our population of interest included patients who received any type of surgical procedures during a hospitalization. The interventions and comparators included seven staffing practices: 1) nurse staffing level; 2) skill mix; 3) RN education; 4) RN experience; 5) work environment characteristics; 6) overtime use; and 7) temporary/agency staff use. Postsurgical outcomes included the following: 1) PCEs, defined as any new onset of myocardial ischemia, dysrhythmias, congestive heart failure, or fluid overload [2]; 2) all-cause mortality; and 3) failure-to-rescue, defined as death following potentially preventable hospital-acquired complications [30,31]. To search the literature, the keywords listed in Table 1 were used. Because keywords related to specific PCEs were too restrictive, we broadened the search by using more general terms (e.g., postsurgical adverse events, nursing-sensitive outcomes) (Table 1). Using these keywords, the first two authors (JBL, LAA) independently searched three electronic databases: CINAHL, PsychInfo, and Medline. Each article retrieved was then independently screened by first reading the title and the abstract and, if necessary, the full text to determine whether a given study met the inclusion criteria. Any disagreements were discussed between the two authors who performed the search and, if necessary, the input of a senior researcher (CMR) was added to resolve any remaining discrepancies. Finally, the reference lists of the included studies were searched to identify any additional relevant studies. We also searched the selected electronic databases to identify any prior or subsequent studies published by the authors of the retrieved articles that could also meet our inclusion criteria.

**Table 1:** Keywords used for the electronic searches.

**Population**	**Interventions / Comparators**	**Outcomes**	**Time**
Postoperativ*^1^ OR surg* OR “surg* patient*” OR operative* AND “acute care hospital*”	“Nurs* staff*” OR “skill mix” OR education mix OR “RN education” OR “registered nurse education” OR overtime OR turnover AND nurs* OR staff* OR RN OR “registered nurse”	“Death” OR “in-hospital death” OR “mortality” OR “30-day mortality” OR “nurs* sensitive outcomes” OR “nurs* sensitive adverse events” OR “adverse* health care event*” OR “postoperative* complication*” OR outcome* OR adverse* OR “failure to rescue” OR mortal*	(since 1996)

^1^The star refers to a truncation, or word stemming, and is a technique that is used to broaden a literature search to include various word endings and spellings.

To be included, studies had to 1) examine the association between at least one nurse staffing practice of interest and at least one of the selected patient outcomes; 2) pertain to a surgical patient population or, when a mixed medical-surgical population was studied, report separate results for surgical and medical patients; 3) be based on a quantitative research design (e.g., cross-sectional, case-control, cohort study); 4) report on objectively measured patient outcomes; 5) use independent data sources to measure the independent and dependent variables; 6) be written in English or French; 7) have been published between January 1, 1996 (the year when the National Academy of Medicine published its landmark report on the adequacy of nurse staffing in hospitals) [32] and August 30, 2018. Studies not meeting these inclusion criteria were excluded.

### Data extraction

Using a standardized data collection tool, the following information was systematically extracted from the retrieved studies by the first author: 1) title, 2) authors, 3) publication and data collection years, 4) study location, 5) study design, 6) surgery type and sample size, 7) independent and dependent variables measured, 8) confounders accounted for in the analyses, 9) data sources, 10) unit of analysis (patient, unit, or hospital), and 11) main study findings. Two other authors (LAA, CMR) validated the extraction to ensure accuracy, and disagreements were resolved through discussions. When data could not be retrieved from the selected articles, their authors were contacted. We contacted five authors and, while all five responded, only four could provide the missing information. The main reason for missing data was that the requested information (e.g., number of patients sampled) was unknown.

### Data analysis

We first present descriptive statistics on the included articles. Then, we provide a qualitative synthesis of their findings. We used *p* < 0.05 as the threshold of statistical significance. Given the methodological heterogeneity of the retrieved studies and since many studies were subcomponents of broader projects also included in this review, no meta-analysis was attempted.

### Study quality appraisal

Four specific design checklists from the Joanna Briggs Institute were used to assess the methodological quality of the retrieved studies. The checklists are: 1) Analytical Cross-Sectional Studies; 2) Cohort Studies; 3) Case-Control Studies; and 4) Quasi-Experimental Studies [33–36]. These checklists each consist of a series of eight design-specific questions related to the clarity with which settings, sampling strategies, and measurement of the independent, dependent, and cofounding variables are described as well as to the appropriateness of the statistical analyses performed. Study quality appraisal was performed independently by two authors (JBL, LAA), and disagreements were resolved through discussions.

## Results

### Study characteristics

**Fig 1:**
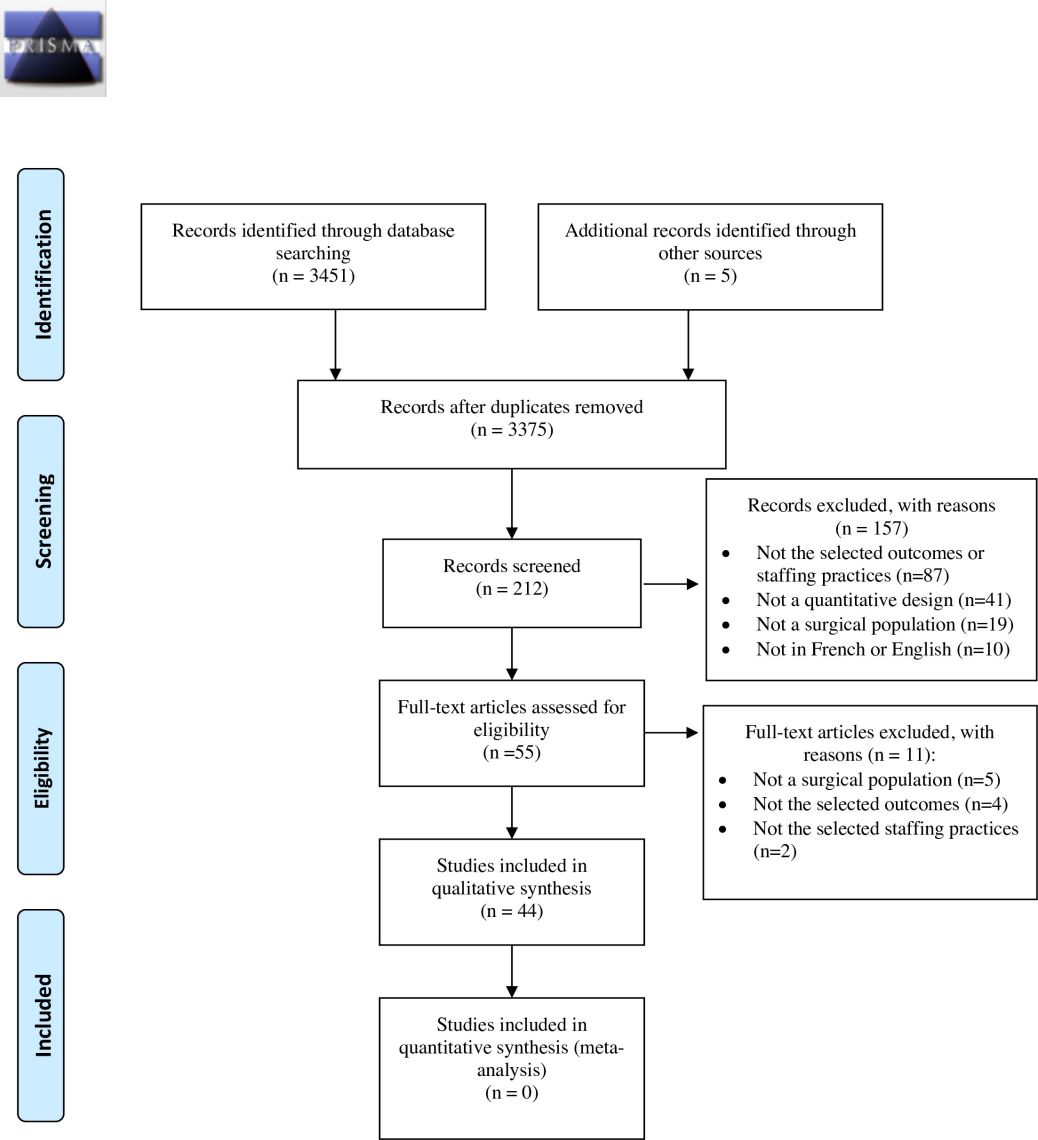
PRISMA flow diagram.

Among the 3,375 potential studies retrieved, 44 articles were included in this systematic review of the literature (Fig 1. PRISMA Flow Diagram.). The primary reasons for excluding studies were because they did not pertain to the selected staffing practices or patient outcomes, or they combined medical and surgical patients in their analyses, even if different outcomes and effect sizes were to be expected (Fig 1) [37].

The characteristics of the included studies are described in S2 Table. These studies were conducted in North America (n = 28, 63.6%), Europe (n = 11, 25.0%), Oceania (n = 3, 6.8%), and Asia (n = 2, 4.5%). They were published between 2001 and 2018 and were based on data initially collected between 1989 and 2009. The median time from data collection to publication was 8 years (range 3 to 16 years). Half (n = 22, 50.0%) of the studies relied on survey data (e.g., nurse, manager or physician surveys) for measuring nurse staffing practices and on administrative data for capturing patient outcomes, whereas the other half measured staffing and outcomes from administrative data only (S2 Table). Most studies (n = 39, 95.1%) relied on cross-sectional designs and used the hospital as their unit of analysis (n = 37, 90.2%). As such, most were multisite investigations involving a median number of 166 hospitals (range: 1–3,485 hospitals). The typical study was based on a median number of 232,432 patients (range: 200–66,100,672 patients). These patients were sampled from different surgical specialties: 1) cardiac (n = 7 studies; 15.9%), 2) vascular (n = 3, 6.8%), 3) oncology (n = 2, 4.5%), or 4) abdominal surgery (n = 2, 4.5%). However, it was more common for several surgical specialties to be combined (n = 29, 65.9%). The most prevalent combinations were 1) general, orthopedic, and vascular surgeries (n = 18, 40.9%) and 2) any type of surgeries (n = 11, 25.0%) (S2 Table).

The most frequently studied staffing practices evaluated were nurse staffing levels (n = 42, 95.5%), RN education (n = 18, 40.9%), work environment characteristics (n = 13, 29.5%), skill mix (n = 8, 18.2%), and RN experience (n = 3 6.8%) (S2 Table). The use of overtime hours was the focus of only one study, and no study pertaining to the use of temporary staff could be retrieved. In addition, 15 studies (35.1%) simultaneously examined the associations of two or more staffing practices on the selected outcomes. The most common combinations were 1) RN staffing levels, RN education, and work environment characteristics (n = 10, 22.7%), 2) RN staffing levels and skill mix (n = 7, 15.9%), and 3) RN staffing levels and RN education (n = 4, 9.1%) (S2 Table). These staffing practices were operationalized in many ways across studies. The sole exception was work environment characteristics, which were consistently assessed using the Practice Environment Scale of the Nursing Work Index or its subscales. (S2 Table) [38].

Great heterogeneity was also observed with regard to outcome measurement (S2 Table). For instance, failure-to-rescue could be operationalized according to different definitions [30,31], and the occurrence of both failure-to-rescue and mortality were ascertained over different time windows (e.g., in-hospital, 30-day or 90-day mortality). Conversely, PCEs were always measured at the time of hospital discharge but from different data sources (e.g., discharge diagnostic codes, chart review). Moreover, they were most often operationalized as a composite of several PCEs, with the most common combinations being 1) cardiac arrest and shock (n = 5, 50.0%) and 2) miscellaneous combinations of cardiac complications (n = 4, 40.0%) (S2 Table). Last, many potential hospital-level confounders (e.g., size, teaching status, technology, location, case mix) and patient-level confounders (e.g., age, sex, comorbidities, type of surgery performed) were adjusted for in the statistical analyses but again, with great heterogeneity across studies (S2 Table).

### Methodological quality assessment

The 44 articles retrieved were of good overall methodological quality given their designs (S3 Table). Specifically, 27 (69.2%) cross-sectional studies satisfied all 8 methodological criteria [20,21,26,30,37,39–60], whereas another 7 (17.9%) studies met 7 of the criteria [24,54–59]. We only found 5 (12.8%) cross-sectional studies that failed to meet two or more quality criteria; the most common methodological issues were an unclear description of the study sample (e.g., number of patients included, type of surgery performed) and an incomplete description of the staffing measures employed [22,23,60–62]. A similar pattern of high methodological quality was observed for the few studies that relied on other designs (S3 Table).

### Literature synthesis

#### Associations between nurse staffing practices and PCEs

We found 10 studies that examined the association between nurse staffing practices and PCEs, either measured alone or in various combinations (Table 2). Most of these studies reported nonsignificant or mixed associations (Table 2). Of note, two studies intended to measure the association between nurse staffing levels and a specific PCE (i.e., fluid overload) [23,62]. However, in both cases, the outcome occurrence was too low, which precluded any statistical analyses (Table 2).

**Table 2:** Nurse staffing practices in association with Postoperative Cardiac Events (PCEs).

Nurse staffing practices	Summary of study findings		
	Significant^1^	Nonsignificant^2^	Mixed^3^
Cardiac arrest and shock			
Staffing (n = 5)		Berney and Needleman [24], McCloskey and Diers [63], Needleman et al. [31], Twigg et al. [64], Van den Heede et al. [48]	
Skill mix (n = 3)		Berney and Needleman [24], McCloskey and Diers [63], Needleman et al. [31]	
Overtime use (n = 1)		Berney and Needleman [24]	
Education (n = 1)		Van den Heede et al. [48]	
Shock or myocardial infarction			
Staffing (n = 1)			Schreuders et al. [19]^4^
Skill mix (n = 1)			Schreuders et al. [19]^4^
Miscellaneous combinations of PCEs/Individual PCEs			
Staffing (n = 3)^5^	Mark et al. [62]^6^	Dimick et al. [65]^7^	Dang et al. [55]

^1^Statistically significant association (p < 0.05) in the expected/hypothesized direction (e.g., higher staffing levels or richer RN skill mix were associated with lower PCE rates).

^2^Nonstatistically significant association (p ≥ 0.05).

^3^Mixed associations refer to both statistically significant (p < 0.05) and nonstatistically significant (p ≥ 0.05) associations reported for the same independent variable.

^4^One statistically significant but inverted association suggested that increasing staffing levels and skill mix were associated with higher odds of PCEs. Other associations with staffing and skill mix were nonsignificant.

^5^Mark et al. [23] is not shown in the table. Fluid overload occurrences were too low, which precluded any statistical analyses.

^6^Fluid overload occurrences were too low, which precluded any statistical analyses. Only the association between miscellaneous combinations of PCE and nurse staffing levels were analyzed.

^7^These authors investigated the associations between RN staffing levels and cardiac arrest and myocardial infarction in separate analyses.

#### Association between nurse staffing practices and mortality

The results of the retrieved studies are summarized by 1) outcome (all-cause mortality vs. failure-to-rescue) and 2) the time-window for outcome assessment (in-hospital vs. 30 days after discharge).

### In-hospital all-cause mortality

We found 17 studies that investigated the association between nurse staffing practices and in-hospital mortality (Table 3). Among these, 16 (94.1%) focused on RN staffing, of which 8 (50.0%) reported nonsignificant associations, 3 (18.8%) reported significant findings, and 5 (31.3%) reported mixed associations (Table 3). Skill mix and RN education were unrelated to in-hospital mortality, whereas more supportive environments were associated with lower in-hospital mortality in only one study (Table 3). Last, one study reported that higher overtime use was significantly associated with lower mortality (Table 3).

**Table 3:** Nurse staffing practices in association with in-hospital mortality.

Nurse staffing practice	Summary of study findings		
	Significant^1^	Nonsignificant^2^	Mixed^3^
Staffing (n = 16)	Berney and Needleman [24], Rafferty et al. [46], Yasunaga et al. [59]	Dimick et al. [65], Hickey et al. [66], Hickey et al. [56], Kiekkas et al. [67], McCloskey and Diers [63], Needleman et al. [31], Newhouse et al. [26], Van den Heede et al. [48]	Diya et al. [40]^4^, Diya et al. [68]^4^, Li et al. [69], Twigg et al. [64]^5^, Van den Heede et al. [49]^4^
Skill mix (n = 4)		Berney and Needleman [24], Hickey et al. [66], McCloskey and Diers [63], Needleman et al. [31]	
Overtime use (n = 1)	Berney and Needleman [24]^6^		
Work environment (n = 1)	Olds et al. [44]		
Education (n = 3)		Newhouse et al. [26], Van den Heede et al. [48]	Van den Heede et al. [49]

^1^Statistically significant association (p < 0.05) in the expected/hypothesized direction (e.g., higher staffing levels or richer RN skill mix were associated with lower mortality rates).

^2^Nonstatistically significant association (p ≥ 0.05).

^3^Mixed associations refer to both statistically significant (p < 0.05) and nonstatistically significant associations reported for the same independent variable.

^4^Nurse staffing was significantly associated with mortality on postoperative units, but not in intensive care units (ICUs). This mixed finding was attributed by the authors to the low of variability of nurse staffing levels in ICUs.

^5^The association was significant when data were aggregated at the hospital level but nonsignificant at nursing unit level.

^6^Statistically significant association, but in the opposite/unexpected direction (i.e., higher overtime use was associated with lower mortality).

### 30-day (or 90-day) mortality

We found 20 studies that examined the associations between nurse staffing practices and 30-day (n = 20) or 90-day (n = 1) mortality (Table 4). The results of these studies contrast sharply with those using in-hospital mortality as the outcome. For instance, among the 16 studies pertaining to RN staffing, 11 (68.8%) reported significant associations, 3 (18.8%) reported mixed findings, and only 2 (12.5%) reported nonsignificant associations (Table 4). Similar patterns also prevailed for studies relating work environment characteristics (87.5% reported significant findings) or RN education (75% reported significant findings) to 30-day mortality (Table 4). Last, both skill mix and RN experience were inconsistently associated with 30-day mortality (Table 4).

**Table 4:** Nurse staffing practices in association with 30-day mortality.

Nurse staffing practice	Summary of study findings		
	Significant^1^	Nonsignificant^2^	Mixed^3^
Staffing (n = 16)	Aiken et al. [51, 52, 53, 70, 71], Ball et al. [39], Carthon et al. [54], Cho et al. [20], Friese et al. [41], Kutney-Lee and Aiken [43], Neff et al. [57]	Elkassabany et al. [60], Halm et al. [72]	Ozdemir et al. [45]^4^, Schreuders et al. [19]^5^, Wiltse Nicely et al. [50]
Skill mix (n = 3)	Aiken et al. [73]	Elkassabany et al. [60]	Schreuders et al. [19]^5^
Work environment (n = 8)	Aiken et al. [52, 53], Cho et al. [20], Friese et al. [41], Neff et al. [57], Rao et al. [47], Wiltse Nicely et al. [50]	Aiken et al. [73]	
Education (n = 12)	Aiken et al. [52, 53, 70, 71], Ball et al. [39], Cho et al. [20], Friese et al. [41], Kendall-Gallagher et al. [42], Kutney-Lee and Aiken [43]	Lane-Fall et al. [74], Wiltse Nicely et al. [50]	Neff et al. [57]
Experience (n = 3)		Aiken et al. [70], Kendall-Gallagher et al. [42]	Lane-Fall et al. [74]

^1^Statistically significant association (p < 0.05) in the expected/hypothesized direction (e.g., higher staffing levels or richer RN skill mix were associated with lower mortality rates).

^2^Nonstatistically significant association (p ≥ 0.05).

^3^Mixed associations refer to both statistically significant (p < 0.05) and nonstatistically significant associations reported for the same independent variable.

^4^This study measured both 30-day and 90-day mortality, but only 30-day mortality was significantly associated with nurse staffing levels.

^5^Two statistically significant but inverted associations suggest that increasing staffing levels and skill mix were associated with higher odds of mortality. Other associations with staffing and skill mix were nonsignificant.

### In-hospital failure-to-rescue

We found 8 studies exploring the associations between nurse staffing practices and in-hospital failure-to-rescue, of which 6 (75.0%) reported significant findings and only 2 (25.0%) reported nonsignificant associations (Table 5). In addition, one study reported that higher overtime use was not significantly associated with in-hospital failure-to-rescue rates (Table 5). Last, among studies pertaining to RN skill mix (n = 3) or RN education (n = 1), none reported significant associations with in-hospital failure-to-rescue (Table 5).

**Table 5:** Nurse staffing practices in association with in-hospital failure-to-rescue.

Nurse staffing practice	Summary of study findings		
	Significant^1^	Nonsignificant^2^	Mixed^3^
Staffing (n = 8)	Berney and Needleman [24], Griffiths et al. [22], Harless and Mark [61], Needleman et al. [31], Rafferty et al. [46], Yasunaga et al. [59]	Twigg et al. [64], Van den Heede et al. [48]	
Skill mix (n = 3)		Berney and Needleman [24], Harless and Mark [61], Needleman et al. [31]	
Overtime use (n = 1)		Berney and Needleman [24]	
Education (n = 1)		Van den Heede et al. [48]	

^1^Statistically significant association (p < 0.05) in the expected/hypothesized direction (e.g., higher staffing levels or richer RN skill mix were associated with lower failure-to-rescue rates).

^2^Nonstatistically significant association (p ≥ 0.05).

^3^Mixed associations refer to both statistically significant (p < 0.05) and nonstatistically significant associations reported for the same independent variable.

### 30-day failure-to-rescue

We found 16 studies exploring the associations between nurse staffing practices and 30-day failure-to-rescue (Table 6). The results of these studies are similar to those observed for 30-day mortality. For instance, among the 13 studies pertaining to RN staffing, 9 (69.2%) reported significant findings, 2 (15.4%) reported mixed associations, and only 2 (15.4%) reported nonsignificant relations (Table 6). More supportive work environments (5 studies, 83.3%) and a higher proportion of RNs educated at the baccalaureate degree level (6 studies, 75.0%) were also consistently associated with lower 30-day failure-to-rescue rates (Table 6). Last, only one study reported that a lower RN skill mix was associated with lower 30-day failure-to-rescue, whereas two studies reported that the RNs’ levels of experience were not (Table 6).

**Table 6:** Nurse Staffing Practices in Association with 30-Day Failure-to-Rescue.

Nurse staffing practice	Summary of study findings		
	Significant^1^	Nonsignificant^2^	Mixed^3^
Staffing (n = 13)	Aiken et al. [51, 52, 53, 70], Carthon et al. [54], Ghaferi et al. [21], Kutney-Lee and Aiken [43], Neff et al. [57], Schreuders et al. [19]	Friese et al. [41], Halm et al. [72]	Sochalski et al. [58], Wiltse Nicely et al. [50]
Skill mix (n = 1)			Schreuders et al. [19]^4^
Work environment (n = 6)	Aiken et al. [53], Friese et al. [41], Neff et al. [57], Wiltse Nicely et al. [50], Rao et al. [47]	Aiken et al. [52]	
Education (n = 8)	Aiken et al. [52, 53, 70], Friese et al. [41], Kendall-Gallagher et al. [42], Kutney-Lee and Aiken [43]	Wiltse Nicely et al. [50]	Neff et al. [57]
Experience (n = 2)		Aiken et al. [70], Kendall-Gallagher et al. [42]	

^1^Statistically significant association (p < 0.05) in the expected/hypothesized direction (e.g., higher staffing levels or richer RN skill mix were associated with lower failure-to-rescue rates.

^2^Nonstatistically significant association (p ≥ 0.05).

^3^Mixed associations refer to both statistically significant (p < 0.05) and nonstatistically significant associations (p ≥ 0.05) reported for the same independent variable.

^4^One statistically significant but inverted association suggested that a lower RN skill mix was associated with lower 30-day failure-to-rescue. Other associations with staffing and skill mix were nonsignificant.

## Discussion

The occurrence of PCEs and death remains high in the postsurgical period despite the availability of preventive measures. This suggests that new risk factors must be identified and addressed. The purpose of this systematic review was to summarize existing evidence regarding one potentially important and modifiable risk factor for PCEs and death: the nurse staffing practices implemented in the postsurgical period.

We found that few studies examined the associations between these staffing practices and PCEs, which suggests that further research is required. Moreover, the results of existing studies were inconsistent. Such inconsistencies are most likely attributable to the important methodological heterogeneity that characterizes this body of research. For instance, we noted that both the independent (e.g., staffing levels, skill mix) and the dependent variables (PCEs, mortality and failure-to-rescue) were operationalized in many ways across studies and measured from several distinct data sources that varied in completeness and accuracy (e.g., surveys, administrative databases). Moreover, we observed that these studies also differed in sample size, in the number of staffing practices measured and in the organizational characteristics (e.g., volume, size, teaching status) accounted for in the analyses, all of which may contribute to explaining the heterogeneous findings across the studies. Moreover, data aggregation at various levels of analysis (i.e., patient, nursing unit, hospital) and reliance on risk adjustment strategies that differ in exhaustiveness and accuracy may have also influenced the likelihood of finding significant and consistent associations. This high degree of methodological heterogeneity, which has been previously observed in the broader field of nurse staffing and patient outcomes research [14–16,75,76], highlights the importance of standardizing methodological approaches in future studies. Greater standardization is also required for generating a robust body of evidence that can support staffing decisions at the bedside.

Alternatively, inconsistent findings across studies could also result from the fact that some staffing practices (e.g., staffing levels) may have little variability on certain types of nursing units (e.g., ICUs typically have similar nurse-to-patient ratios, whereas such ratios may vary more extensively across general surgical units) [40,49,68]. To address this issue, alternative and more refined measures of staffing adequacy that vary across units should be explored in future studies (e.g., percentage of unmet nursing care needs, workload adequacy). Moreover, greater variability could also have been generated by the differences in study design (i.e., varying from hospital-level cross-sectional studies to patient-level longitudinal investigations).

Interestingly, we observed that PCEs were most commonly operationalized as a composite outcome of several distinct PCEs (e.g., cardiac arrest and shock). This methodological choice was attributed to the low incidence rate of PCEs in some of the reviewed studies, which precluded their analysis as separate outcomes [23,62]. While pooling PCEs into a composite outcome may allow for statistical inferences, future research is required to determine the validity and clinical utility of this approach and to elucidate the mechanisms that likely explain any observed associations between nurse staffing practices and PCEs.

In parallel, future research is also needed to identify more accurate and efficient methods for measuring PCEs. Indeed, we found that most of the reviewed studies relied on discharge diagnostic codes, which are well known for their low sensitivity and positive predictive value for identifying adverse events such as PCEs [77–79]. In addition, while manual chart review is the reference standard in this area of research, it is labor-intensive, time-consuming, and costly. As a consequence, studies that relied on this method were often based on small samples of patients and consequently underpowered for detecting any significant associations between nurse staffing practices and PCEs [79]. Among the potential alternatives to discharge diagnostic codes and manual chart review, the recent development and validation of novel methods for measuring adverse events directly from electronic health record data and clinical narratives appears promising [80–83].

Another important finding of this systematic review is that adequate nurse staffing levels, a higher proportion of RNs educated at the baccalaureate degree level, and more supportive work environments were related to lower rates of both 30-day mortality and failure-to-rescue. One possible explanation for these associations is that RNs are responsible for providing important interventions as part of their surveillance of the patients under their care, such as patient teaching, discharge planning or care coordination across settings [60,84], the effect of which may only become apparent after discharge. Moreover, recent studies have suggested that, under suboptimal staffing circumstances, nurses will deliberately omit (ration) these care processes to prioritize more critical medical treatments and interventions, which may impact outcomes after discharge [84,85]. In addition, it is plausible that more supportive work environments and the usage of a greater proportions of RNs educated at the baccalaureate degree level may influence the RNs’ decision-making processes and priority-setting or allow them to exploit the full extent of their scope of practice, all of which could explain why these staffing practices were associated with better patient outcomes [39,82–84]. While there is emerging evidence for these associations, further research is required to better elucidate the mechanisms by which nurse staffing levels, RN education and work environment characteristics are linked to patient mortality and failure-to-rescue.

Moreover, we noted that higher nurse staffing, a higher proportion of RNs educated at the baccalaureate degree level, and more supportive work environments were more consistently related to failure-to-rescue than to mortality (especially when these outcomes were measured at the time of hospital discharge). All-cause mortality has been criticized by several nursing scholars for its lack of sensitivity to nursing care [14,37], whereas failure-to-rescue, or deaths resulting from potentially preventable complications, has stronger theoretical underpinnings with nursing interventions, notably through the concept of nurse surveillance [14,17,37,41]. As such, given that staffing levels, a higher proportion of RNs educated at the baccalaureate degree level, and more supportive work environments are important to effective nurse surveillance, it is possible that failure-to-rescue is simply more sensitive than mortality to variations in these nurse staffing practices.

Interestingly, most of the reviewed studies were based on cross-sectional designs and typically analyzed hospital-level administrative data. While this approach is also commonly used in the broader field of nurse staffing and patient outcomes research [14–16,37], the validity of its findings has been questioned for two important and interrelated reasons. Cross-sectional designs preclude the assessment of the temporal sequence linking an exposure to its associated outcome [79], whereas hospital-level data imprecisely allocate nursing resources to individual patients [84,86]. Therefore, to strengthen this body of evidence, there is a strong need for longitudinal studies conducted at the patient level of analysis.

Moreover, across the reviewed studies, scant attention has been given to other important staffing practices, such as skill mix and the use of overtime hours or temporary nursing staff. In addition, few studies have examined the simultaneous association of these (and other) staffing practices with the occurrence of PCEs, mortality or failure-to-rescue. Given that multiple staffing practices are typically employed simultaneously by managers on any given nursing unit and shift in an attempt match available resources with the patients’ requirements for nursing care, estimating the simultaneous effects of these practices on patient outcomes is an important next step in the investigation [87]. Such studies are required to assist managers in identifying which staffing practices are associated with greater benefits to patients.

Finally, some limitations of this systematic review must be acknowledged. First, although we used a comprehensive list of keywords, it is possible, as in any systematic review, that some important studies were omitted. We therefore recommend that our work be periodically updated and expanded. Second, some of the reviewed studies used subsamples of larger projects also included in this review. While this approach has the merit of being exhaustive, it may also have somewhat amplified some of our conclusions. Third, high methodological heterogeneity and the overlapping nature of several studies precluded a formal meta-analysis. Indeed, pooling heterogenous/overlapping studies would bias the estimated effect of a given staffing practice on outcomes [67]. Last, limitations of this systematic review also include those of the reviewed studies, such as inaccurate and incomplete coding of patient characteristics and outcomes in administrative databases, low response rates on surveys measuring nurse staffing practices, incomplete risk-adjustment, and poor matching of each staffing practice to the actual patients cared for, all of which could have influenced the conclusions of this systematic review.

## Conclusions

We found evidence that nurse staffing levels, a higher proportion of RNs educated at the baccalaureate degree level and more supportive work environments are cross-sectionally associated with a lower risk of 30-day mortality and failure-to-rescue. Moreover, among the few studies that pertained to PCEs, inconsistent results were observed. To strengthen this body of research and support evidence-based staffing decisions at the bedside, there is a strong need for patient-level longitudinal studies. Such studies are also required to determine whether less frequently investigated staffing practices, such as overtime or temporary/agency staff use, are related to the occurrence of PCEs, mortality or failure-to-rescue.

## Supporting information

S1 Table PRISMA checklist. (DOC)

S2 Table Characteristics of the studies included in the systematic review. (DOCX)

S3 Table Methodological quality assessment. (DOCX)

## References

[1] Weiser TG, Haynes AB, Molina G, Lipsitz SR, Esquivel MM, Uribe-Leitz T, et al Size and distribution of the global volume of surgery in 2012. Bull World Health Organ. 2016;94: 201F–209F. 26966331 10.2471/BLT.15.159293 PMC4773932

[2] Sellers D, Srinivas C, Djaiani G. Cardiovascular complications after non-cardiac surgery. Anaesthesia. 2018;73 Suppl 1: 34–42. 29313903 10.1111/anae.14138

[3] Agency for Healthcare Research and Quality. AHRQ national scorecard on hospital-acquired conditions: updated baseline rates and preliminary results 2014–2017; 2019 [cited 2019 Apr 19] Available from: https://www.ahrq.gov/sites/default/files/wysiwyg/professionals/quality-patient-safety/pfp/hacreport-2019.pdf

[4] Sabate S, Mases A, Guilera N, Canet J, Castillo J, Orrego C, et al Incidence and predictors of major perioperative adverse cardiac and cerebrovascular events in non-cardiac surgery. Br J Anaesth. 2011;107: 879–890. 10.1093/bja/aer268 21890661

[5] Devereaux PJ, Sessler DI. Cardiac complications in patients undergoing major noncardiac surgery. N Engl J Med. 2015;373: 2258–2269. 10.1056/NEJMra1502824 26630144

[6] Botto F, Alonso-Coello P, Chan MT, Villar JC, Xavier D, Srinathan S, et al Myocardial injury after noncardiac surgery: a large, international, prospective cohort study establishing diagnostic criteria, characteristics, predictors, and 30-day outcomes. Anesthesiology. 2014;120: 564–578. 10.1097/ALN.0000000000000113 24534856

[7] Puelacher C, Lurati Buse G, Seeberger D, Sazgary L, Marbot S, Lampart A, et al Perioperative myocardial injury after noncardiac surgery: incidence, mortality, and characterization. Circulation. 2018;137: 1221–1232. 10.1161/CIRCULATIONAHA.117.030114 29203498

[8] Greenberg JW, Lancaster TS, Schuessler RB, Melby SJ. Postoperative atrial fibrillation following cardiac surgery: a persistent complication. Eur J Cardiothorac Surg. 2017;52: 665–672. 10.1093/ejcts/ezx039 28369234

[9] Bessissow A, Khan J, Devereaux PJ, Alvarez-Garcia J, Alonso-Coello P. Postoperative atrial fibrillation in non-cardiac and cardiac surgery: an overview. J Thromb Haemost. 2015;13 Suppl 1: S304–S312. 26149040 10.1111/jth.12974

[10] Fayad A, Ansari MT, Yang H, Ruddy T, Wells GA. Perioperative diastolic dysfunction in patients undergoing noncardiac surgery is an independent risk factor for cardiovascular events: a systematic review and meta-analysis. Anesthesiology. 2016;125: 72–91. 10.1097/ALN.0000000000001132 27077638

[11] Khan NA, Quan H, Bugar JM, Lemaire JB, Brant R, Ghali WA. Association of postoperative complications with hospital costs and length of stay in a tertiary care center. J Gen Intern Med. 2006;21: 177–180. 10.1111/j.1525-1497.2006.00319.x 16606377 PMC1484655

[12] Acheampong D, Guerrier S, Lavarias V, Pechman D, Mills C, Inabnet W, et al Risk factors contributing to cardiac events following general and vascular surgery. Ann Med Surg (Lond). 2018;33: 16–23. 10.1016/j.amsu.2018.08.001 30147870 PMC6105747

[13] Duceppe E, Parlow J, MacDonald P, Lyons K, McMullen M, Srinathan S, et al Canadian cardiovascular society guidelines on perioperative cardiac risk assessment and management for patients who undergo noncardiac surgery. Can J Cardiol. 2017;33: 17–32. 10.1016/j.cjca.2016.09.008 27865641

[14] Audet LA, Bourgault P, Rochefort CM. Associations between nurse education and experience and the risk of mortality and adverse events in acute care hospitals: a systematic review of observational studies. Int J Nurs Stud. 2018;80: 128–146. 10.1016/j.ijnurstu.2018.01.007 29407346

[15] Driscoll A, Grant MJ, Carroll D, Dalton S, Deaton C, Jones I, et al The effect of nurse-to-patient ratios on nurse-sensitive patient outcomes in acute specialist units: a systematic review and meta-analysis. Eur J Cardiovasc Nurs. 2018;17: 6–22. 10.1177/1474515117721561 28718658

[16] Stalpers D, de Brouwer BJ, Kaljouw MJ, Schuurmans MJ. Associations between characteristics of the nurse work environment and five nurse-sensitive patient outcomes in hospitals: a systematic review of literature. Int J Nurs Stud. 2015;52: 817–835. 10.1016/j.ijnurstu.2015.01.005 25655351

[17] Kutney-Lee A, Lake ET, Aiken LH. Development of the hospital nurse surveillance capacity profile. Res Nurs Health. 2009;32: 217–228. 10.1002/nur.20316 19161172 PMC2906760

[18] Aiken LH, Clarke SP, Sloane DM. Hospital staffing, organization, and quality of care: cross-national findings. Nurs Outlook. 2002;50: 187–194. 12386653 10.1067/mno.2002.126696

[19] Schreuders LW, Bremner AP, Geelhoed E, Finn J. The relationship between nurse staffing and inpatient complications. J Adv Nurs. 2015;71: 800–812. 10.1111/jan.12572 25414059

[20] Cho E, Sloane DM, Kim EY, Kim S, Choi M, Yoo IY, et al Effects of nurse staffing, work environments, and education on patient mortality: an observational study. Int J Nurs Stud. 2015;52: 535–542. 10.1016/j.ijnurstu.2014.08.006 25213091 PMC4286441

[21] Ghaferi AA, Osborne NH, Birkmeyer JD, Dimick JB. Hospital characteristics associated with failure to rescue from complications after pancreatectomy. J Am Coll Surg. 2010;211: 325–330. 10.1016/j.jamcollsurg.2010.04.025 20800188

[22] Griffiths P, Jones S, Bottle A. Is "failure to rescue" derived from administrative data in England a nurse sensitive patient safety indicator for surgical care? Observational study. Int J Nurs Stud. 2013;50: 292–300. 10.1016/j.ijnurstu.2012.10.016 23195407

[23] Mark BA, Harless DW. Nurse staffing and post-surgical complications using the present on admission indicator. Res Nurs Health. 2010;33: 35–47. 10.1002/nur.20361 20014218

[24] Berney B, Needleman J. Impact of nursing overtime on nurse-sensitive patient outcomes in New York hospitals, 1995–2000. Policy Polit Nurs Pract. 2016;7: 87–100. 10.1177/1527154406291132 16864629

[25] Hart P, Davis N. Effects of nursing care and staff skill mix on patient outcomes within acute care nursing units. J Nurs Care Qual. 2011;26: 161–168. 10.1097/NCQ.0b013e3181efc9cb 20706144

[26] Newhouse RP, Johantgen M, Pronovost PJ, Johnson E. Perioperative nurses and patient outcomes-mortality, complications, and length of stay. AORN J. 2005;81: 508–528. 10.1016/s0001-2092(06)60438-9 15799504

[27] Silber JH, Romano PS, Rosen AK, Wang Y, Even-Shoshan O, Volpp KG. Failure-to-rescue: comparing definitions to measure quality of care. Med Care. 2007;45: 918–925. 10.1097/MLR.0b013e31812e01cc 17890988

[28] Liberati A, Altman DG, Tetzlaff J, Mulrow C, Gotzsche PC, Ioannidis JP, et al The PRISMA statement for reporting systematic reviews and meta-analyses of studies that evaluate health care interventions: explanation and elaboration. J Clin Epidemiol. 2009;62: e1–e34. 10.1016/j.jclinepi.2009.06.006 19631507

[29] Riva JJ, Malik KMP, Burnie SJ, Endicott AR, Busse JW. What is your research question? An introduction to the PICOT format for clinicians. J Can Chiropr Assoc. 2012;56: 167–171. 22997465 PMC3430448

[30] Silber JH, Williams SV, Krakauer H, Schwartz JS. Hospital and patient characteristics associated with death after surgery. A study of adverse occurrence and failure to rescue. Med Care. 1992;30: 615–629. 10.1097/00005650-199207000-00004 1614231

[31] Needleman J, Buerhaus P, Mattke S, Stewart M, Zelevinsky K. Nurse-staffing levels and the quality of care in hospitals. N Engl J Med. 2002;346: 1715–1722. 10.1056/NEJMsa012247 12037152

[32] Institute of Medicine (US) Committee on the Adequacy of Nursing Staff in Hospitals and Nursing Homes. Nursing staff in hospitals and nursing homes: is it adequate? In: Wunderlich GS, Sloan F, Davis CK, editors. Washington, DC: National Academies Press; 1996. 25121200

[33] Moola S, Munn Z, Tufanaru C, Aromataris E, Sears K, Sfetcu R, et al Checklist for analytical cross sectional studies; 2017 [cited 2018 Aug 15]. Available from: https://joannabriggs.org/sites/default/files/2019-05/JBI_Critical_Appraisal-Checklist_for_Analytical_Cross_Sectional_Studies2017_0.pdf

[34] Moola S, Munn Z, Tufanaru C, Aromataris E, Sears K, Sfetcu R, et al Checklist for case control studies; 2017 [cited 2018 Aug 15]. Available from: https://joannabriggs.org/sites/default/files/2019-05/JBI_Critical_Appraisal-Checklist_for_Case_Control_Studies2017_0.pdf

[35] Moola S, Munn Z, Tufanaru C, Aromataris E, Sears K, Sfetcu R, et al Checklist for cohort studies; 2017 [cited 2018 Aug 15]. Available from: https://joannabriggs.org/sites/default/files/2019-05/JBI_Critical_Appraisal-Checklist_for_Cohort_Studies2017_0.pdf

[36] Moola S, Munn Z, Tufanaru C, Aromataris E, Sears K, Sfetcu R, et al Checklist for quasi-experimental studies (non-randomized experimental studies); 2017 [cited 2017 Aug 15]. Available from: https://joannabriggs.org/sites/default/files/2019-05/JBI_Quasi-Experimental_Appraisal_Tool2017_0.pdf

[37] Kane RL, Shamliyan TA, Mueller C, Duval S, Wilt TJ. The association of registered nurse staffing levels and patient outcomes: systematic review and meta-analysis. Med Care. 2007;45: 1195–1204. 10.1097/MLR.0b013e3181468ca3 18007170

[38] Lake ET. Development of the practice environment scale of the nursing work index. Res Nurs Health. 2002;25: 176–188. 10.1002/nur.10032 12015780

[39] Ball JE, Bruyneel L, Aiken LH, Sermeus W, Sloane DM, Rafferty AM, et al Post-operative mortality, missed care and nurse staffing in nine countries: a cross-sectional study. Int J Nurs Stud. 2018;78: 10–15. 10.1016/j.ijnurstu.2017.08.004 28844649 PMC5826775

[40] Diya L, Lesaffre E, Van den Heede K, Sermeus W, Vleugels A. Establishing the relationship between nurse staffing and hospital mortality using a clustered discrete-time logistic model. Stat Med. 2010;29: 778–785. 10.1002/sim.3756 20213720

[41] Friese CR, Lake ET, Aiken LH, Silber JH, Sochalski J. Hospital nurse practice environments and outcomes for surgical oncology patients. Health Serv Res. 2008;43: 1145–1163. 10.1111/j.1475-6773.2007.00825.x 18248404 PMC2517272

[42] Kendall-Gallagher D, Aiken LH, Sloane DM, Cimiotti JP. Nurse specialty certification, inpatient mortality, and failure to rescue. J Nurs Scholarsh. 2011;43: 188–194. 10.1111/j.1547-5069.2011.01391.x 21605323 PMC3201820

[43] Kutney-Lee A, Aiken LH. Effect of nurse staffing and education on the outcomes of surgical patients with comorbid serious mental illness. Psychiatr Serv. 2008;59: 1466–1469. 10.1176/appi.ps.59.12.1466 19033176 PMC2596648

[44] Olds DM, Aiken LH, Cimiotti JP, Lake ET. Association of nurse work environment and safety climate on patient mortality: a cross-sectional study. Int J Nurs Stud. 2017;74: 155–161. 10.1016/j.ijnurstu.2017.06.004 28709013 PMC5695880

[45] Ozdemir BA, Sinha S, Karthikesalingam A, Poloniecki JD, Pearse RM, Grocott MP, et al Mortality of emergency general surgical patients and associations with hospital structures and processes. Br J Anaesth. 2016;116: 54–62. 10.1093/bja/aev372 26675949

[46] Rafferty AM, Clarke SP, Coles J, Ball J, James P, McKee M, et al Outcomes of variation in hospital nurse staffing in English hospitals: cross-sectional analysis of survey data and discharge records. Int J Nurs Stud. 2007;44: 175–182. 10.1016/j.ijnurstu.2006.08.003 17064706 PMC2894580

[47] Rao AD, Kumar A, McHugh M. Better nurse autonomy decreases the odds of 30-day mortality and failure to rescue. J Nurs Scholarsh. 2017;49: 73–79. 10.1111/jnu.12267 28094907 PMC5460530

[48] Van den Heede K, Sermeus W, Diya L, Clarke SP, Lesaffre E, Vleugels A, et al Nurse staffing and patient outcomes in Belgian acute hospitals: cross-sectional analysis of administrative data. Int J Nurs Stud. 2009;46: 928–939. 10.1016/j.ijnurstu.2008.05.007 18656875 PMC2700208

[49] Van den Heede K, Lesaffre E, Diya L, Vleugels A, Clarke SP, Aiken LH, et al The relationship between inpatient cardiac surgery mortality and nurse numbers and educational level: analysis of administrative data. Int J Nurs Stud. 2009;46: 796–803. 10.1016/j.ijnurstu.2008.12.018 19201407 PMC2856596

[50] Wiltse Nicely KL, Sloane DM, Aiken LH. Lower mortality for abdominal aortic aneurysm repair in high-volume hospitals is contingent upon nurse staffing. Health Serv Res. 2013;48: 972–991. 10.1111/1475-6773.12004 23088426 PMC3651774

[51] Aiken LH, Clarke SP, Sloane DM, Sochalski J, Silber JH. Hospital nurse staffing and patient mortality, nurse burnout, and job dissatisfaction. JAMA. 2002;288: 1987–1993. 10.1001/jama.288.16.1987 12387650

[52] Aiken L, Clarke S, Sloane D, Lake E, Cheney T. Effects of hospital care environment on patient mortality and nurse outcomes. J Nurs Adm. 2008;38: 223–229. 10.1097/01.NNA.0000312773.42352.d7 18469615 PMC2586978

[53] Aiken LH, Cimiotti JP, Sloane DM, Smith HL, Flynn L, Neff DF. Effects of nurse staffing and nurse education on patient deaths in hospitals with different nurse work environments. Med Care. 2011;49: 1047–1053. 10.1097/MLR.0b013e3182330b6e 21945978 PMC3217062

[54] Carthon JM, Kutney-Lee A, Jarrin O, Sloane D, Aiken LH. Nurse staffing and postsurgical outcomes in black adults. J Am Geriatr Soc. 2012;60: 1078–1084. 10.1111/j.1532-5415.2012.03990.x 22690984 PMC3376011

[55] Dang D, Johantgen ME, Pronovost PJ, Jenckes MW, Bass EB. Postoperative complications: does intensive care unit staff nursing make a difference? Heart Lung. 2002;31: 219–228. 12011813 10.1067/mhl.2002.122838

[56] Hickey PA, Gauvreau K, Jenkins K, Fawcett J, Hayman L. Statewide and national impact of California's staffing law on pediatric cardiac surgery outcomes. J Nurs Adm. 2011;41: 218–225. 10.1097/NNA.0b013e3182171b2e 21519208

[57] Neff DF, Cimiotti J, Sloane DM, Aiken LH. Utilization of non-US educated nurses in US hospitals: implications for hospital mortality. Int J Qual Health Care. 2013;25: 366–372. 10.1093/intqhc/mzt042 23736834 PMC3723304

[58] Sochalski J, Konetzka R, Zhu J, Volpp K. Will mandated minimum nurse staffing ratios lead to better patient outcomes? Med Care. 2008;46: 606–613. 10.1097/MLR.0b013e3181648e5c 18520315

[59] Yasunaga H, Hashimoto H, Horiguchi H, Miyata H, Matsuda S. Variation in cancer surgical outcomes associated with physician and nurse staffing: a retrospective observational study using the Japanese diagnosis procedure combination database. BMC Health Serv Res. 2012;12: 129 10.1186/1472-6963-12-129 22640411 PMC3405470

[60] Elkassabany NM, Passarella M, Mehta S, Liu J, Neuman MD. Hospital characteristics, inpatient processes of care, and readmissions of older adults with hip fractures. J Am Geriatr Soc. 2016;64: 1656–1661. 10.1111/jgs.14256 27351297 PMC4988892

[61] Harless DW, Mark BA. Nurse staffing and quality of care with direct measurement of inpatient staffing. Med Care. 2010;48: 659–663. 10.1097/MLR.0b013e3181dbe200 20548254

[62] Mark BA, Harless DW, Berman WF. Nurse staffing and adverse events in hospitalized children. Policy Polit Nurs Pract. 2007;8: 83–92. 10.1177/1527154407303499 17652626

[63] McCloskey BA, Diers DK. Effects of New Zealand’s health reengineering on nursing and patient outcomes. Med Care. 2005;43: 1140–1146. 10.1097/01.mlr.0000182549.85761.cd 16224308

[64] Twigg D, Duffield C, Bremner A, Rapley P, Finn J. The impact of the nursing hours per patient day (NHPPD) staffing method on patient outcomes: a retrospective analysis of patient and staffing data. Int J Nurs Stud. 2011;48: 540–548. 10.1016/j.ijnurstu.2010.07.013 20696429

[65] Dimick JB, Swoboda SM, Pronovost PJ, Lipsett PA. Effect of nurse-to-patient ratio in the intensive care unit on pulmonary complications and resource use after hepatectomy. Am J Crit Care. 2001;10: 376–382. 11688604

[66] Hickey P, Gauvreau K, Connor J, Sporing E, Jenkins K. The relationship of nurse staffing, skill mix, and Magnet recognition to institutional volume and mortality for congenital heart surgery. J Nurs Adm. 2010;40: 226–232. 10.1097/NNA.0b013e3181da3f71 20431457

[67] Kiekkas P, Sakellaropoulos GC, Brokalaki H, Manolis E, Samios A, Skartsani C, et al Association between nursing workload and mortality of intensive care unit patients. J Nurs Scholarsh. 2008;40: 385–390. 10.1111/j.1547-5069.2008.00254.x 19094155

[68] Diya L, Van den Heede K, Sermeus W, Lesaffre E. The relationship between in-hospital mortality, readmission into the intensive care nursing unit and/or operating theatre and nurse staffing levels. J Adv Nurs. 2012;68: 1073–1081. 10.1111/j.1365-2648.2011.05812.x 21883408

[69] Li X, Bowman SM, Smith TC. Effects of registered nurse staffing level on hospital-acquired conditions in cardiac surgery patients: a propensity score matching analysis. Nurs Outlook. 2016;64: 533–541. 10.1016/j.outlook.2016.05.002 27311745

[70] Aiken LH, Clarke SP, Cheung RB, Sloane DM, Silber JH. Educational levels of hospital nurses and surgical patient mortality. JAMA. 2003;290: 1617–1623. 10.1001/jama.290.12.1617 14506121 PMC3077115

[71] Aiken LH, Sloane DM, Bruyneel L, Van den Heede K, Griffiths P, Busse R, et al Nurse staffing and education and hospital mortality in nine European countries: a retrospective observational study. Lancet. 2014;383: 1824–1830. 10.1016/S0140-6736(13)62631-8 24581683 PMC4035380

[72] Halm M, Peterson M, Kandels M, Sabo J, Blalock M, Braden R, et al Hospital nurse staffing and patient mortality, emotional exhaustion, and job dissatisfaction. Clin Nurse Spec. 2005;19: 241–254. 16179855 10.1097/00002800-200509000-00007

[73] Aiken LH, Sloane D, Griffiths P, Rafferty AM, Bruyneel L, McHugh M, et al Nursing skill mix in European hospitals: cross-sectional study of the association with mortality, patient ratings, and quality of care. BMJ Qual Saf. 2017;26: 559–568. 10.1136/bmjqs-2016-005567 28626086 PMC5477662

[74] Lane-Fall MB, Ramaswamy TS, Brown SES, He X, Gutsche JT, Fleisher LA, et al Structural, nursing, and physician characteristics and 30-day mortality for patients undergoing cardiac surgery in pennsylvania. Crit Care Med. 2017;45: 1472–1480. 10.1097/CCM.0000000000002578 28661969 PMC5561002

[75] Park SH, Blegen MA, Spetz J, Chapman SA, De Groot HA. Comparison of nurse staffing measurements in staffing-outcomes research. Med Care. 2015;53: e1–e8. 10.1097/MLR.0b013e318277eb50 23222530

[76] Brennan CW, Daly BJ, Jones KR. State of the science: the relationship between nurse staffing and patient outcomes. West J Nurs Res. 2013;35: 760–794. 10.1177/0193945913476577 23444060

[77] Rodrigo-Rincon I, Martin-Vizcaino MP, Tirapu-Leon B, Zabalza-Lopez P, Abad-Vicente FJ, Merino-Peralta A. Validity of the clinical and administrative databases in detecting post-operative adverse events. Int J Qual Health Care. 2015;27: 267–275. 10.1093/intqhc/mzv039 26082462

[78] Romano PS, Mull HJ, Rivard PE, Zhao S, Henderson WG, Loveland S, et al Validity of selected AHRQ patient safety indicators based on VA national surgical quality improvement program data. Health Serv Res. 2009;44: 182–204. 10.1111/j.1475-6773.2008.00905.x 18823449 PMC2669628

[79] Rochefort CM, Buckeridge DL, Abrahamowicz M. Improving patient safety by optimizing the use of nursing human resources. Implement Sci. 2015;10: 89 10.1186/s13012-015-0278-1 26071752 PMC4465738

[80] Rochefort CM, Verma AD, Eguale T, Lee TC, Buckeridge DL. A novel method of adverse event detection can accurately identify venous thromboembolisms (VTEs) from narrative electronic health record data. J Am Med Inform Assoc. 2015;22: 155–165. 10.1136/amiajnl-2014-002768 25332356 PMC4433368

[81] Shiner B, Neily J, Mills PD, Watts BV. Identification of inpatient falls using automated review of text-based medical records. J Patient Saf. 2016: 1 10.1097/PTS.0000000000000088 27331601

[82] Chapman AB, Mowery DL, Swords DS, Chapman WW, Bucher BT. Detecting evidence of intra-abdominal surgical site infections from radiology reports using natural language processing. AMIA Annu Symp Proc. 2017;2017: 515–524. 29854116 PMC5977582

[83] Tvardik N, Kergourlay I, Bittar A, Segond F, Darmoni S, Metzger MH. Accuracy of using natural language processing methods for identifying healthcare-associated infections. Int J Med Inform. 2018;117: 96–102. 10.1016/j.ijmedinf.2018.06.002 30032970

[84] Griffiths P, Recio-Saucedo A, Dall'Ora C, Briggs J, Maruotti A, Meredith P, et al The association between nurse staffing and omissions in nursing care: a systematic review. J Adv Nurs. 2018;74: 1474–1487. 10.1111/jan.13564 29517813 PMC6033178

[85] Schubert M, Ausserhofer D, Desmedt M, Schwendimann R, Lesaffre E, Li B, et al Levels and correlates of implicit rationing of nursing care in Swiss acute care hospitals—a cross sectional study. Int J Nurs Stud. 2013;50: 230–239. 10.1016/j.ijnurstu.2012.09.016 23084600

[86] Needleman J, Buerhaus P, Pankratz VS, Leibson CL, Stevens SR, Harris M. Nurse staffing and inpatient hospital mortality. N Engl J Med. 2011;364: 1037–1045. 10.1056/NEJMsa1001025 21410372

[87] Manojlovich M, Sidani S, Covell CL, Antonakos CL. Nurse dose: linking staffing variables to adverse patient outcomes. Nurs Res. 2011;60: 214–220. 10.1097/NNR.0b013e31822228dc 21691239

