# Supplementary material for: Are hospital nurse staffing practices associated with postoperative cardiac events and death? A systematic review
Source: PLoS One. 2019 Oct 17;14(10):e0223979. doi: 10.1371/journal.pone.0223979 (PMC6797123; doi:10.1371/journal.pone.0223979)
Supplement: S2 Table — (DOCX) [file pone.0223979.s002.docx]

**S2 Table.**

| # | Authors | Publication year (data collection years) | Location | Study design | Data sources | Sample characteristics | Study variables | | | Level of analysis | Main results |
| --- | --- | --- | --- | --- | --- | --- | --- | --- | --- | --- | --- |
|  |  |  |  |  |  |  | **Independent variables** | **Dependent variables** | **Confounders** |  |  |
|  | Aiken, Clarke, Sloane, Sochalski & Silber | 2002 (1998-1999) | USA | Cross-sectional, multisite | Nurse survey, patient discharge abstract, Pennsylvania Department of Health Hospital Questionnaire and AHA annual survey | 232,342 patients between the age of 20 and 85 years general surgical, orthopedic, or vascular procedure from 168 hospitals center | Nurse staffing: mean RN-patient ratio on the last shift | 30-day mortality  30-day FTR | 1- Patient characteristics: age, sex, surgery type, nature of the hospital admission and comorbidities.  2- Hospital characteristics: hospital size, teaching status and technology level. | Hospital | Nurse staffing is significantly associated to mortality (OR 1.07 95% CI 1.03-1.12) and failure-to-rescue (OR 1.07 95% CI 1.02-1.11). |
|  | Aiken, Clarke, Chenung, Sloane & Silber | 2003 (1998-1999) | USA | Cross-sectional, multisite | Nurse survey, patient discharge abstract, Pennsylvania Department of Health Hospital Questionnaire and AHA annual survey | 232,342 patients between the age of 20 and 85 years who underwent general surgical, orthopedic, or vascular procedure from 168 hospitals center | Nurse staffing: mean RN-patient ratio on the last shift  Nurse education: proportion of RN holding a bachelor’s degree or higher  Nurse experience: years of experience working as a RN | 30-day mortality  30-day FTR | 1- Patient characteristics: age, sex, nature of the hospital admission, transfer from another hospital, surgery type, and comorbidities.  2- Hospital characteristics: hospital size, teaching status and technology level.  3- Medical staffing: presence of board-certified surgeon | Hospital | Each 10% increase in the proportion of nurse with higher degrees decrease the risk of mortality and of FTR (OR 0.95 95% IC 0.91-0.99)  Nurses’ years of experience were not found to be a significant predictor of mortality or FTR.  RN staffing was also significantly associated to mortality (1.14 95% CI 1.08-1.19) and FTR (1.11 95% CI 1.06-1.16). |
|  | Aiken, Clarke, Sloane, Lake & Cheney | 2008 (1998- 1999) | USA | Cross-sectional, multisite | Nurse survey, patient discharge abstract, Pennsylvania Department of Health Hospital Questionnaire and AHA annual survey | 232,342 patients between the age of 20 and 85 years who underwent general surgical, orthopedic, or vascular procedure from 168 hospitals center | Nurse staffing: mean RN-patient ratio on the last shift  Nurse education: proportion of RN holding a bachelor’s degree or higher  Patient care environment: practice environment scales of the Nursing Work Index (PES-NWI) | 30-day mortality  30-day FTR | 1- Patient characteristics: age, sex, nature of the hospital admission, transfer from another hospital, surgery type, and comorbidities  2- Hospital characteristics: hospital size, teaching status and technology level  3- Medical staffing: presence of board-certified surgeon | Hospital | Individually, nurse staffing (OR 1.08 95%IC 1.03 – 1.13), nurse education (OR 0.94 95% IC 0.90 – 0.97), and care environment (OR 0.91 95% IC 0.85 – 0.97) were significantly associated to 30-day mortality. Also, nurse staffing (OR 1.08 95%IC 1.03 – 1.13), nurse education (OR 0.93 95% IC 0.89 – 0.97), and care environment (OR 0.91 95% IC 0.85 – 0.98) were significantly associated to 30-day FTR.  Jointly, the association is still significant for all of RN characteristics, except for care environment and 30-day FTR. |
|  | Aiken, Cimiotti, Sloane, Smith, Flynn & Neff | 2011 (2005-2008) | USA | Cross-sectional, multisite | Nurse survey, patient discharge abstract, and AHA annual  survey | 1,262,120 patients between the age of 20 and 85 years who underwent general surgical, orthopedic, or vascular procedure from 665 hospitals center | Nurse staffing: mean RN-patient ratio on the last shift  Nurse education: proportion of RN holding a bachelor’s degree or higher  Patient care environment: practice environment scales of the Nursing Work Index (PES-NWI) | 30-day mortality  30-day FTR | 1- Patient characteristics: age, sex, transfer from another hospital, surgery type, and comorbidities  2- Hospital characteristics: hospital size, teaching status and technology level | Hospital | For mortality, significant associated were observed with nurse staffing (OR 1.039 95%IC 1.016 – 1.063), nurse education (OR 0.958 95%IC 0.937 – 0.980) and care environment (OR 0.926 95%IC 0.898 – 0.955)  For FTR, significant associated were observed with nurse staffing (OR 1.039 95%IC 1.016 – 1.063), nurse education (OR 0.956 95%IC 0.935 – 0.978) and care environment (OR 0.925 95%IC 0.897 – 0.954) |
|  | Aiken, Sloane, Bruyneel, den Heede, Griffiths, Busse, Diomidous, … & Sermeus (RN4CAST consortium) | 2014 (2007-2010) | Belgium, England, Finland, Ireland, the Netherland, Norway, Spain, Sweden and Switzerland | Cross-sectional, multisite | Nurse survey and government administrative data (hospital characteristics and patient abstract) | 422,730 patients 50 years old and older, who underwent general surgical, orthopedic, or vascular procedure from 300 hospitals center | Nurse staffing: mean RN-patient ratio on the last shift  Nurse education: proportion of RN holding a bachelor’s degree or higher | 30-day mortality | 1- Patient characteristics: age, sex, admission type, surgery type, and comorbidities  2- Hospital characteristics: location, size, teaching status and technology level  3- Nursing characteristics: practice environment scales of the Nursing Work Index (PES-NWI) | Hospital | Both nurse staffing (OR 1.068 IC 95% 1.031 – 1.106) and nurse education (0.929 IC 95% 0.886 – 0.973) were significantly associated with mortality |
|  | Aiken, Sloane, Griffiths, Rafferty, Bruyneel, McHugh, … & Sermeus (RN4CAST consortium) | 2017 (2007-2010) | Belgium, England, Finland, Ireland, Spain, Switzerland | Cross-sectional, multisite | Nurse survey and government administrative data (hospital characteristics and patient abstract) | 275,519 patients 50 years old and older, who underwent general surgical, orthopedic, or vascular procedure from 243 hospitals center | Nurse skill mix: average proportion of RN divide by the total of direct care personnel of all qualification | 30-day mortality | 1- Patient characteristics: age, sex, admission type, surgery type, and comorbidities  2- Hospital characteristics: size, teaching status, technology level and practice environment scales of the Nursing Work Index (PES-NWI)  3- nurse staffing: average total staffing by 25 patients and average proportion of nurse holding a bachelor’s degree or higher | Hospital | There was no statistically significant association between PES-NWI and mortality. PES-NWI was not added to the fully adjusted model.  An significant association between nurse skill mix and mortality was observed (OR 0.89 IC95% 0.80-0.98) |
|  | Ball, Bruyneel, Aiken, Sermeus, Sloane, Rafferty, … & Griffiths (RN4CAST consortium) | 2018 (2007-2010) | Belgium, England, Finland, Ireland, the Netherland, Norway, Spain, Sweden and Switzerland | Cross-sectional, multisite | Nurse survey and government administrative data (hospital characteristics and patient abstract) | 422,730 patients 50 years old and older, who underwent general surgical, orthopedic, or vascular procedure from 300 hospitals center | Nurse staffing: average RN-patient ratio  Nurse education: average proportion of RN holding a bachelor’s degree or higher  Missed care: average percentage of care activity reported as missed by hospital nurse | 30-day mortality | 1- Patient characteristics: age, sex, admission type, surgery type, and comorbidities.  2- Hospital characteristics: location, size, teaching status, technology level and practice environment scales of the Nursing Work Index (PES-NWI) | Hospital | When nurse staffing and education are included in the bayesian model in addition to missed care, the direct relationship between nurse staffing and patient mortality is no longer significant showing that missed care mediate the association. However the effect of nurse education remained significant. |
|  | Berney & Needleman | 2006 (1995-2000) | USA | Cross-sectional, multisite | Government and insurance databases | 729,027 for cardiac arrest and shock, 26,675 FTR to 913,750 patients for mortality who underwent a major surgery from 161 hospitals. | Nurse staffing: RN hour per patient day (adjusted for Nursing Intensity Weights)  Nurse skill mix: percentage of RN hour on the sum of RN hours and LPN hours  Nurse overtime: percentage of RN overtime | In-hospital mortality  In-hospital FTR  Postoperative cardiac arrest and shock | 1- Patient characteristics: age, race, admission type, payer, and comorbidities.  2- Hospital characteristics: hospital size, unionization, margin.  3- Medical staffing: percentage of resident and intern per bed, percentage of Foreign Medical Graduates residents | Hospital | More overtime is associated with a decrease in mortality only (OR 0.984 *p*< 0.01).  RN hour per patient day is associated to FTR (OR 0.979  *p*< 0.01) and mortality (OR 0.969  *p*< 0.001).  None of the association with nurse skill mix or with cardiac arrest and shock were significant. |
|  | Carthon, Kutney-Lee, Jarrìn, Sloane & Aiken | 2012 (2005-2006) | USA | Cross-sectional, multisite | Nurse survey,  Patient discharge abstract,  and AHA annual  survey | 548,397 patients, black and white adults, who underwent general surgical, orthopedic, or vascular procedure from 599 hospitals | Nurse staffing: average RN-patient ratio on the last shift | 30-day mortality  30-day FTR | 1- Patient characteristics: age, race, sex, neighborhood Socioeconomic Summary Index score, transfer from another hospital, insurance type, admission diagnostic, and comorbidities.  2- Hospital characteristics: size, teaching status, population density and technology level. | Hospital | After adjusting for hospital, nursing and patient characteristics, the odds of 30-day mortality for black patients were not significantly different from those of white patients. The odds of death increase for all patients by factor of 1.03 (95%IC 1.01 -1.05) with each additional patient in the average nurse workload.  In another model, a significant interaction was found between race and nurse staffing for the outcome of 30-day mortality, such that black patient had higher odds of death (OR 1.10 95%IC 1.03 – 1.18) that white (OR 1.03 95%IC 1.01 – 1.05). A similaire effet size was found for FTR (Black: OR 1.10 95%IC 1.03 – 1.17 and white: OR 1.03 95%IC 1.01 – 1.06). |
|  | Cho, Sloane, Kim, Kim, Choi, Yoo, … & Aiken | 2015 (2008- 2009) | South Korea | Cross-sectional, multisite | Nurse survey and government administrative data (hospital characteristics and patient abstract) | 76,036 patients, 50 years old and older, who underwent general surgical, orthopedic, or vascular procedure from 14 high-technology and teaching hospital | Nurse staffing: average RN-patient ratio on the last shift  Nurse education: average proportion of RN holding a bachelor’s degree or higher  Patient care environment: Korean Practice Environment Scales of the Nursing Work Index (PES-NWI) | 30-day mortality | 1- Patient characteristics: age, sex, admission type, transfer from another hospital, surgery type, and comorbidities.  2- Hospital characteristics: size and location | Hospital | A marginal association was observed between nurse staffing and mortality (OR 1.05 95% IC 1.00 - 1.10).  Nurse work environment (OR 0.52 95% IC 0.31 - 0.88) and nurse education were significantly associated to mortality (OR 0.91 95% IC 0.83 – 0.99). |
|  | Dang, Johantgen, Pronovost, Jenckes & Bass | 2002 (1994-1996) | USA | Cross-sectional, multisite | Physician ICU directors with nurse managers survey and Government administrative data | 2,606 patients undergoing abdominal aortic surgery, 30 years old or older, from 38 ICU that responded to the survey on nurse staffing | Nurse staffing: RN-patient ratio coded as “low-intensity staffing” (1:3 or greater on the day and night shift), “medium-intensity staffing” (1:3 or greater on either the day or night shift but not both) and “high-intensity staffing” (1:2 or fewer on the day and night shifts) | Acute myocardial infarction, cardiac arrest, cardiac complication after a procedure | 1- Patient characteristics: Age, sex, race, rupture or unruptured aorta and comorbidities.  2- Hospital characteristics: hospital volume of abdominal aortic surgery per year, ICU size and use of critical paths | Ward | Patient cared for on units with medium-intensity staffing were more likely to have a cardiac complication when compared to high-intensity staffing units (OR 1.78 IC95% 1.16 – 2.72). This association is not significant for patient cared for on units with low-intensity compared to high-intensity units (OR 1.34 IC95% 0.82 – 2.17). |
|  | Dimick, Swoboda, Pronovost & Lipsett | 2001 (1994-1998) | USA | Cross-sectional, multisite | Physician ICU directors survey and Government administrative data | 569 patients undergoing hepatectomy, 18 years old or older and from 33 hospitals | Nurse staffing: RN-patient ratio coded as “more ICU nurses” (1:1 or 1:2) or “fewer ICU nurses” (1:3 or more) | acute myocardial infarction, cardiac arrest, cardiac complication  In-hospital mortality | 1- Patient characteristics: age, sex, nature of admission, type of surgery and comorbidities.  2- Hospital characteristics: hospital and surgeon volume | Ward | None of the cardiac complication was significantly associate to nurse staffing in ICU (cardiac arrest OR 0.6 *p=*0.90 and myocardial infarction OR 6.6 *p*=0.27, cardiac complication not reported).  ICU nurse staffing was not associate to in-hospital mortality (OR 0.49 IC95% 0.18 – 1.29). |
|  | Diya, Lesaffre, Van den Heede, Sermus & Vleugels | 2010 (2003) | Belgium | Cross-sectional, multisite | Government administrative data | 9,054 patient between 20 and 85 years old underwent elective coronary artery bypass graft or heart valve procedure from 28 hospitals | Nurse staffing: RN level in ICU and in postoperative units  Nursing intensity: Intensity of nursing care needed in ICU and postoperative units | In-hospital mortality | 1- Patient characteristics: age, sex, type of surgery and comorbidities (risk of mortality).  2- Hospital characteristics: yearly hospital volume of cardiac surgery, average of ICU staff and average of postoperative units staff | Hospital | High staffing levels in general unit is associated with lower risk of mortality (OR, 0.389; 95% CI, 0.114-0.871).  No association were significant between nursing intensity and mortality. |
|  | Diya, Van den Heede, sermeus & Lesaffre | 2012 (2003) | Belgium | Cross-sectional, multisite | Government  administrative data | 9,054 patient between 20 and 85 years old underwent elective coronary artery bypass graft or heart valve procedure from 28 hospitals | Nurse staffing:  RN level in ICU and in postoperative units  Nursing intensity: Intensity of nursing care needed in ICU and postoperative units | ICU mortality and mortality after readmission into the ICU and/or operating theatre | 1- Patient characteristics: age, sex, type of surgery and comorbidities (risk of mortality).  2- Hospital characteristics: yearly hospital volume of cardiac surgery, average of ICU staff and average of postoperative unit staff | Hospital | A interaction between volume and ICU nurse staffing are significantly associated with in-hospital mortality.  No association was observed with staffing and nursing intensity with mortality. |
|  | Elkassabany, Passarella, Mehta, Liu & Neuman | 2016 (2007-2009) | USA | Cross-sectional, multisite | Medicare Provider Analysis and review files (insurance data) | 458,526 Medicare beneficiaries, 65 years old and older and who underwent hip fracture surgery from 3485 hospitals | Nurse staffing: ratio of full-time employee RN and LPN to hospital bed  Nurse skill mix: ratio of full-time RN and LPN to all full-time employee nurses. | 30-day mortality | 1-Patient characteristics: age, sex, race, fracture type, procedure type, nursing home residence and comorbidity | Hospital | After adjustment for participant factors, the odds of death at 30 day was not significant for both nurse staffing (OR 0.99 95%IC 0.98 – 1) and nurse skill mix (OR 0.9 95%IC 0.78 – 1.04). |
|  | Friese, Lake, Aiken, Silber & Sochalski | 2008 (1998-1999) | USA | Cross-sectional, multisite | Nurse survey, hospital administrative data, and AHA annual survey | 25,957 patients undergoing surgical intervention for cancer (head and neck, esophagus, colon-rectum, pancreas, lung, ovary, prostate and endometrium) in 164 hospitals. | Nurse staffing: mean RN-to-patient ratio on the last shift  Nurse education: proportion of RN holding a bachelor’s degree or higher  Patient care environment: practice environment scales of the Nursing Work Index (PES-NWI) | 30-day mortality  30-day FTR | 1- Patient characteristics: age, sex, race, nature of the hospital admission, transfer from another hospital, cancer type/duration/stage and comorbidities.  2- Hospital characteristics: size, teaching status, technology level and National Cancer Institute recognition. | Hospital | Significant predictors of 30-day mortality included poorest nurse staffing (OR 1.41 IC95% 1.03 - 1.91) and unfavorable nurse practice environments (OR 1.37 IC95% 1.07 – 1.76). An increase proportion of nurses with bachelor’s degree or higher was associated with a decreased odd of dying in 30-day (OR 0.46 IC95% 0.21 – 0.98).  Unfavorable practice environment (OR 1.48 IC95% 1.07 – 2.03) and nursing education (OR 0.37 IC95% 0.17 – 0.80) were significant predictors of FTR. Nurse staffing was not significant predictor of FTR. |
|  | Ghaferi, Osborne, Birkmeyer & Dimick | 2010 (2000-2006) | USA | Cross-sectional, multisite | Nationwide Inpatient Sample (NIS) from the Agency for Healthcare Research and Quality (AHRQ) and American Hospital Association (AHA) | 8,862 patients undergoing pancreatectomy from 672 hospitals | Nurse staffing: RN hour per patient day  hospital characteristics: Teaching status, hospital technology, hospital size and average daily census. | 30-day FTR | 1- Patient characteristics: Demographic characteristics and comorbidities. | Hospital | In univariate analysis, nurse to patient ratio was significantly associate to FTR (OR 8.3 IC95% 4.7 – 14.6) and explained 17.0% of the proportion of FTR.  When combined to other hospital characteristics, all characteristics are still significant associated to FTR (OR 6.6 IC95% 3.7 – 11.9). |
|  | Griffiths, Jones & Bottle | 2013 (1997-2009) | England | Cross-sectional, multisite | National Health Service (NHS) Commissioning Data Sets (CDS), Dr Foster Intelligence | 66,100,672 surgical admission defined by AHRQ to 146 hospital | Nurse staffing: FTE RN per bed.  Skill mix: RN per doctor  RN stability: percentage of RN in post during 2008 to 2009. | Failure to rescue (FTR-A): death among surgical inpatient with serious treatable complications.  Failure to rescue (FTR-L): patient with abnormal long hospital stays. | 1- Hospital characteristics: teaching status, number of community-based general medical practitioners, proportion of deaths occurring in hospital, number of hospital discharges and location of the hospital in London and percentage of patient with a FTR, bed occupancy, percentage of patient with a comorbidity, average occupancy number of bed, professionally qualified clinical staff per bed. | Hospital | FTR-A is significantly associate to nurse per doctor (RR 1.04 IC95% 1.03 – 1.06) and to RN stability (RR 1.04 IC95% 1.03 – 1.06). Full-time equivalent RN per bed was associated to FTR-A (RR 0.94 IC95% 0.93 - 0.95) but was not included in the multiple regression model due to multicollinearity.  FTR-L is significantly associate to RN stability (RR 0.97 IC95% 0.96 – 0.99). RN per doctor (RR1.06 IC95% 1.05- 1.06) and Full-time equivalent RN per bed (RR10.8 IC95% 1.08 – 1.09) were significantly associate to FTR-L in univariate analysis but were not included in the multiple regression model. |
|  | Halm, Peterson, Kandels, Sabo, Blalock, Braden, … Topham | 2005 (2002) | USA | Cross-sectional, Unisite | Nurse survey and Hospital’s database | 2,709 patients between the age of 20 and 85 years who underwent general surgical, orthopedic, or vascular procedure from one hospital | Nurse staffing: mean RN- patient ratio by nursing unit | 30-day mortality  30-day FTR | Patient characteristics: age, emergency department admission, circulatory MDC and number of comorbidity | Ward | 30-day mortality and FTR were not significantly associate to nurse staffing. No multivariable analysis was tried. |
|  | Harless and Mark | 2010 (1996-2001) | USA | Cross-sectional, multisite | AHA Annual survey, Area files, InterStudy data, OSHPD annual disclosure report | 485,980 surgical admission from 283 hospitals | Nurse staffing:  RN FTE per 1000 inpatient day  Nurse skill mix:  LVN FTE per 1000 inpatient day  Aide FTE per 1000 inpatient day | In-hospital mortality (excluded from this systematic review because authors combined medical and surgical population for this analysis)  In-hospital FTR | 1- Patient characteristics: age, sex, diagnostic, admission type, admission source.  2-Hospital characteristics: market characteristics, technology level, location, teaching status | Hospital | Nurse staffing was significantly associate to FTR only for the highest staffing level *p* < 0.05 (above 50^th^ percentile). Other staffing levels (LVN and aides) were not significantly associate to FTR. |
|  | Hickey, Gauvrewau, Connor, Sporing & Jenkins | 2010 (2005-2006) | USA | Cross-sectional, multisite | Pediatric Health Information System (PHIS), National Association of Children's Hospitals, Related Institution (NACHRI), Staffing Program (CPSP) database and American Nurses Credentialing Center (ANCC) | 21,885 cases of surgical repair of a congenital heart defect, from 2005 to 2006, younger than 18 years old from 38 hospitals | Nurse staffing: ICU RN worked hours per patient day  Nurse skill mix: ICU percentage of RN among all clinical assistant and non-RN | In-hospital mortality | 1- Patient characteristics: RACHS-1 score: including presence of specific diagnosis, age, combinations of cardiac procedures, prematurity, major non-cardiac structural anomaly and combination of cardiac procedure during the admission.  2- Hospital characteristics: magnet recognition and cardiac procedure volume | Hospital | None of the nursing characteristics (nurse staffing OR 0.88 95% IC 0.72 – 1.08 and nurse skill mix OR 1.02 95% IC 0.88 – 1.18) was associate with mortality. |
|  | Hickey, Gauvreau, Jenkins, Fawcett & Hayman | 2011 (2002-2006) | USA | Cross-sectional, multisite | Utilization Project Kids' inpatient Database and AHA Annual survey | 4,414 cases of congenital heart surgery from 2002 and 2006, younger than 18 years old from 14 hospitals. | Nurse staffing: FTE RN and ratio FTE RN per staffed bed | In-hospital mortality | 1- Patient characteristics: RACHS-1 score: including presence of specific diagnosis, age, combinations of cardiac procedures, prematurity, major non-cardiac structural anomaly and combination of cardiac procedure during the admission. | Hospital | Nurse staffing characteristics (FTE RN OR 1.04 95% IC 0.98 – 1.11 and FTE RN per staffed bed OR 1.13 95% IC 0.76 – 1.78) were not associate to mortality. |
|  | Kendall-Gallagher, Aiken, Sloane & Cimiotti | 2011 (2005-2006) | USA | Cross-sectional, multisite | Nurse survey,  hospital  discharge abstract data,  and AHA annual  survey | 1,283,241 patients, 21 years old or older, who underwent general surgical, orthopedic, or vascular procedure from 652 hospitals | Nurse education: percentage of RN holding a bachelor’s degree or higher and percentage of RN with a certification | 30-day mortality  30-day FTR | 1- Patient characteristics: age sex, diagnostic, surgery type and comorbidities.  2- Hospital characteristics: size, teaching status, technology level and location.  3-Nurse staffing: RN years of experience | Hospital | When nurse education, certification and experience were estimate simultaneously, only nurse education were significantly associate to mortality (OR 0.94 *p*<0.001) and FTR (0.93  *p*<0.001).  When considering percentage of AD or diploma nurse who are certified and percentage of BSN that are certified, analysis shows only a significant effect between percentage of BSN nurse with certification (OR 0.93  *p*<0.01). |
|  | Kekkas, Sakelllaropoulos, Brokalaki, Manolis, Samios, Skartsani & Baltopoulos | 2008 (2005-2006) | Greece | Cross-sectional, unisite | Prospective collect | 200 surgical patients (37.6% cardiac surgery, trauma (19.7%, abdominal surgery 16.25, neurosurgery 11.9%) from one ICU. | Nurse staffing: patient care demand (TISS-28 score)/ total RN) average daily | Mortality during ICU stay | 1- Patient characteristics: acute physiology score, age and comorbidity | Ward | Compared to peak low-exposure group, peak medium-exposure OR 1.36 95% IC 0.50 -1 3.73) and peak high-exposure group (OR 1.94 95% IC 0.76 – 4.94) were not significantly associate to mortality in ICU.  The same analysis was tried with the median exposure (low, medium (OR 1.08 95% IC 0.37 – 3.10) and high OR 1.48 95% IC 0.57 – 3.84) and was not significantly associate to mortality. |
|  | Kutney-Lee & Aiken | 2008 (1998-1999) | USA | Cross-sectional, multisite | Nurse survey, patient discharge abstract, and AHA annual survey | 228,433 patients between the age of 20 and 85 years who underwent general surgical, orthopedic, or vascular procedure from 157 hospitals. | Nurse staffing: mean RN-patient ratio on the last shift  Nurse education: proportion of RN holding a bachelor’s degree or higher | 30-day mortality  30-day FTR | 1- Patient characteristics: age, sex, race, surgery type, admission source, insurance status and comorbidities.  2- Hospital characteristics: size, teaching status, board certification of surgeon and technology level. | Hospital | Nurse staffing (OR 1.05 95% IC 1.01 – 1.09) and more than 40% BSN (OR 0.80 95% IC 0.67 – 0.094) were significantly associated to 30-day mortality. For 30-day FTR, the association with nurse staffing (OR 1.06 95% IC 1.02 – 1.10) and more than 40% BSN (OR 0.52 95% IC 0.28 – 0.96) were also significant.  Significant Interaction were found between mental illness and nurse staffing in association with mortality and FTR. No interaction were found between education and mental illness. |
|  | Lane-Fall, Ramaswamy, Brown, Gutsche, Fleisher & Neuman | 2017 (2009-2011) | USA | Cross-sectional, multisite | Manager survey and government database (patient and hospital characteristics) | 29,449 cardiac surgery patients of 18 years old and older from 43 ICU | Nurse education: proportion of RN with a BSN estimated as “less than 1/3”, “between 1/3 and 2/3” or “greater that 2/3”.  Nurse experience: proportion of RN with <2 years experiences in ICU estimated as “ < 1/3 more experienced” and “> 1/3 less experienced”.  Nurse shit length: number of hours by shift. | 30-day mortality | 1- Patient characteristics: age, race, sex, Charleson comorbidity index, insurance type, admission source and surgery type | Hospital | In multivariate model, none of the nurse staffing characteristics was significantly associate to 30-day mortality after a heart surgery.  Stratifying for patients admitted for emergent procedures shows a association between 30-day mortality and less experienced nurses (OR 1.25 IC95% 1.01 – 1.54). |
|  | Li, Bowman & Smith | 2016 (2009-2011) | USA | Cross-sectional, multisite | Nationwide Inpatient Sample (Healthcare Cost and Utilization Project) | 1,845,750 matched cardiac surgery patient of 18 years old and older from 1,887 hospitals | Nurse staffing: RN hour per patient day (above and below median) | In-hospital mortality following pressure ulcers (stage III/IV), catheter-associated urinary tract infection and central line-associated blood stream infection. | 1- Patient characteristics: age, sex, race, admission on weekend, elective admission, admitted to another facility, admission year and comorbidities.  2- Hospital characteristics: location, ownership, size and teaching status. | Hospital | Higher RN staffing was associated with lower mortality after central line-associated blood stream infection (OR 0.80 95% IC 0.63- 0.93) and catheter-associated urinary tract infection (OR 0.95 95% IC 0.92 – 0.97) compared with patient receiving care in low-staffing hospitals. |
|  | Mark, Harless & Berman | 2007 (1996-2001) | USA | Coss-sectional, multisite | AHA Annual survey, Area Ressource Files, InterStudy data, OSHPD annual disclosure report | 14 years old and younger patient from 286 hospitals | Nurse staffing: RN hour per patient day | In-hospital mortality and FTR (excluded; done on medical surgical inpatient combined)  Postoperative cardiopulmonary complications (other than myocardial infarction) and fluid overload | 1-Patient characteristics: RDScale score (age, sex, admission type and comorbidities).  2- Hospital characteristics: location, number of pediatric bed, number of pediatric inpatient day, presence of pediatric and neonate intensive care, year of hospitalization, location, teaching status and licensed vocational nurses per patient day and unlicensed personnel hour per patient day. | Hospital | Fluid overload could not be reliably analyzed because it occurred so infrequently at the vast majority of hospitals.  Postoperative cardiopulmonary complications at 25^th^ percentile of nurse staffing, a 1-hour increase in staffing resulted in a 4.4% reduction in these complications, whereas at the 75^th^ percentile, the reduction (3.1%) was smaller but still significant. |
|  | Mark and Harless | 2009 (1996-2001) | USA | Cross-sectional, multisite | AHA Annual survey, Area Ressource Files, InterStudy data, OSHPD annual disclosure report and non-public OSPHD patient-level file | 485,980 surgical admissions from 283 hospitals | Nurse staffing: RN hour per patient day, LVN per patient day and aides hour per patient day | Fluid overload | 1-Patient characteristics: COC score (age, sex,, comorbidities, discharge status and length of stay).  2- Hospital characteristics: technology services (Saidin index) and hospital market area (Herfindahl index) | Hospital | There were too few instances in which the expected count for fluid overload exceeded 15, no analyze were tried. |
|  | McCloskey and Diers | 2005 (1989-2000) | New Zealand | Longitudinal, multisite | National Minimum Dataset (NMDS) and Nursing Workforce Dataset (NWD) | 1,042,587 surgical admissions statewide | Nurse staffing: number of nursing FTEs/ 1000 patients, number of nursing hours worked/1000 patients  Nurse Skill mix: percentage of RN hours among all nursing personnel | Shock and cardiac arrest  In-hospital mortality (variation between 1989 and 2000) | none | State | The nursing workforce analysis showed decreases in the number of nurse FTEs and their associated hours worked, and an increase in skill mix from 1992 to 2000. during this period, shock increase by 16% and surgical mortality stayed equal (relation insignificant). |
|  | Needleman, Buerhaus, Mattke, Stewart and Zelevinsky | 2002 (1997) | USA | Cross-sectional, multisite | AHA annual survey, administrative government data | 1,104,659 surgical patients from 790 hospitals | Nurse staffing: hours of nursing care per inpatient-day  Nursing skill mix: proportion of hours provided by RN | In-hospital morality  In-hospital FTR  Shock and cardiac arrest | 1- Patient characteristics: age, sex, patient’s diagnosis-related group, primary health insurer, admission type and comorbidities.  2- Hospital characteristics: size, teaching status and location. | Hospital | A greater number of RN hours per patient day was associated with lower rate of FTR and a greater number of licensed-nurse-hours per day was also associated with lower FTR (incidence-rate ratio 0.98 95%IC 0.97 – 1.00).  There was no association between in-hospital morality or shock and cardiac arrest and the proportion of registered-nurse-hours, the number of registered-nurse-hours per day or the number of licensed-nurse-hours per day. |
|  | Neff, Cimotti, Sloane and Aiken | 2013 (2005-2007) | USA | Cross-sectional, multisite | Nurse survey,  hospital  discharge abstract data  and AHA annual  survey | 1,295,179 patients between the age of 19 and 90 years who underwent general surgical, orthopedic, or vascular procedure from 665 hospitals | Nurse staffing: mean RN- patient ratio on the last shift  Nurse education: percentage of non-US-educated RN per hospital  Patient care environment based on the practice environment scales of the Nursing Work Index (PES-NWI) | 30-day mortality  30-day FTR | 1- Patient characteristics: age, sex, transfer status, surgery type, and comorbidities.  2- Hospital characteristics: hospital size, teaching status and technology level.  3- Nurse staffing: proportion of nurse holding a bachelor’s degree or higher, proportion of medical-surgical nurse and proportion of intensive care unit nurse. | Hospital | Nurse staffing (OR 1.03 95% IC 1.00 – 1.06) and practice environment (OR 0.93 95% IC 0.90 – 0.96) are significantly associated with mortality.  Nurse staffing (OR 1.03 95%IC 1.00 – 1.05) and practice environment (OR 0.93 95%IC 0.90 – 0.96) are significantly associated with FTR.  The main effect of the percentage of non-educated nurses is not significant with morality and FTR.  The relationship between the proportion of non-US-educated nurses and mortality and FTR is significant when analyzed with his interaction with nurse staffing. |
|  | Newhouse, Johantgen, Pronovost and Johnson | 2005 (2000-2002) | USA | Cross-sectional, multisite | Survey of Maryland perioperative directors or managers, patient discharge data (HSCRC) | 1,894 patient who underwent abdominal aneuvrym repair and aortic-iliac-femoral bypass from 32 hospitals | Nurse staffing: Number of RN per case, RN agency use,  Nurse education: CNOR certification  Nurse work shift: 24 hours staffing  Performance of multidisciplinary code drill. | In-hospital mortality | 1- Patient characteristics: age, sex, comorbidity, rupture, admitting code and extubation in OR  2-Hospital characteristics: hospital volume | Hospital | Only RN agency use was significantly associated to mortality (OR 0.67 95%IC 0.56 – 0.80). All other RN characteristics were not significantly associate to mortality. |
|  |  |  |  |  |  |  |  |  |  |  |  |
|  | Olds, Aiken, Cimiotti and Lake | 2017 (2005-2007) | USA | Cross-sectional, multisite | Nurse survey, patient discharge abstract and AHA Annual Survey | 852,974 patients between the age of 18 and 89 years who underwent general surgical, orthopedic, or vascular procedure from 600 hospitals center | Patient care environment based on the practice environment scales of the Nursing Work Index (PES-NWI)  Hospital safety climate: perception of nurse about safety | In-hospital mortality | 1- Patient characteristics: age, sex, race, surgery type and comorbidities.  2- Hospital characteristics: hospital size, teaching status and technology level.  3-Nurse characteristics: hospital proportion of nurse respondant from medical surgical and intensive care unit and nurse staffing, education. | Hospital | In separate model, both patient care environment (OR 0.919 95%IC 0.888 – 0.950) and hospital safety climate (OR 0.923 95%IC 0.893 – 0.956) were significantly associated to mortality.  In the fully adjusted model, patient care environment was significant (OR 0.94 95%IC 0.888 – 0.996) and safety climate lost statistical significance. |
|  | Ozdemir, Karthikesalingam, Poloniecki, Pearse, Grocott, Thompson and Holt | 2016 (2005-2010) | England | Cross-sectional, multisite | Hospital Episode Statistics records, Office of National Statistics (ONS) Registry, Department of Health and Health and Social Care Information Centre | 294,602 patient who underwent emergency general surgical procedure (appendicectomy, hernia repair, colorectal, laparotomy, peptic ulcer surgery) from 156 hospitals | Nurse staffing: total RN staffing (categorical variable) | 30-day mortality  90-day mortality  30-day FTR | 1-Patient characteristics: age, sex, social deprivation indices, stratification by years of discharge, weekend vs weekday admission and comorbidities.  2- Hospital characteristics: total critical beds, operating theatres, MRI usage, CT usage, total ultrasound, total fluoroscopies  3- Medical staffing: total doctors, total doctors in general surgery, consultant doctors in general surgery, senior house officers in general surgery, house officers in general surgery, total senior house officers and total house officers, teaching status. | Hospital | In the fully adjusted model, patient hospitalized in hospital with lower staffing(OR 1.070 95%IC 1.08 – 1.134) and middle staffing (OR 1.086 95%IC 1.029 – 1.148) had higher odds of 30-day mortality then patient hospitalized in the highest total nurse staffing.  No association was found between nurse staffing and 90-day mortality.  Failure to rescues was not analyzed with nurse staffing variable. |
|  | Rafferty, Clarke, Coles, Ball, James, McKee and Aiken | 2007 (1998-1999) | England | Cross-sectional, multisite | Nurse survey, patient discharge abstract and hospital administrative database | 118,752 patients between 20 and 85 years olds, who underwent general, orthopedic, vascular surgical procedure in 30 hospitals | Nurse staffing: mean RN-patient ratio on the last shift | In-hospital mortality  In-hospital FTR | Patient characteristics: age, mode of admission, major diagnostic category  Hospital characteristics: size, teaching status and technology | Hospital | Patient in hospital in the upper quartile (where to nurse had the heaviest patient loads) were 26% (OR 1.26 95%IC 1.09 – 1.46) more likely to die overall and 29% (1.29 95%IC 1.12 -1.49) more likely to die.  The association between FTR and staffing was only significant for the second (OR 1.20 95% IC 1.03 -1.40) and forth quartile (OR 1.29 95% IC 1.12 – 1.49). |
|  | Rao, Kumar and McHugh | 2017 (2005-2007) | USA | Cross-sectional, multisite | Nurse survey, patient discharge abstract and AHA Annual Survey | 1,222,870 patients between the age of 18 and 85 years who underwent general surgical, orthopedic, or vascular procedure from 570 hospitals center | Patient care environment: nurse perception of autonomy (Nursing Workload Index-Revised) | 30-day mortality  30-day FTR | 1- Patient characteristics: age, sex, surgery type and comorbidity  2-Hospital characteristics: size, teaching status, technology level  3- Nurse characteristics: RN-patient ratio, proportion of nurse holding a bachelor’s degree or higher | Hospital | Each additional point on nurse autonomy was associated with approximately 19% lower odds of 30-day mortality (p<0.05) and 17% lower odds of FTR (p<0.05). |
|  | Schreuders, Bremner, Geelhoed & Finn | 2015 (2004-2008) | Australia | Longitudinal, multisite | Health Corporate Network, WA Data Linkage Branch and Health Information Network | 6,333 hospitalizations with admission to a high-intensity unit following a theatre procedure from 3 hospitals | Nurse staffing: RN hours per patient day  Nurse skill mix: proportion of total nursing hours provided by RN | 30-day mortality  30-day FTR  Shock or myocardial infarction | 1- Patient characteristics: age, sex, relative decile socioeconomic status, month of admission, admission type, inter-hospital transfer, nursing units which of the three hospitals they attended and comorbidities. | Patient | When skill mix was low, NHPPD was only significantly associated to FTR (OR 0.17 95%IC 0.06 – 0.49). Increase in NHPPD decrease the odd of FTR.  When skill mix is mid, NHPPD is significantly associated to 30-day mortality (OR 1.16 95%IC 1.08 -1.23). A increase in NHPPD increase the odd of mortality.  When skill mix is high, NHPPD is significantly associated to shock and myocardial infarction (OR 1.09 95%IC 0.1.03 - 1.15) and 30-day mortality (OR: 1.11 95%IC 1.05 – 1.17). Increase in NHPPD increase the odds of both complications. |
|  | Sochalski, Konetzka, Zhu & Volpp | 2008 (1993-2001) | USA | Cross-sectional/ longitudinal, multisite | California Office of Statewide Health Planning and Development's (OSHPD), Medicare case-mix index data files, Census Bureau data, OSHPD - patient discharge records and annual wage index files from Centers for Medicare and Medicaid Services | 109,066 patient who underwent major general, orthopedic or vascular surgical procedure from 343 hospitals | Nurse staffing: RN hours per patient day and RN + LVN hours per patient day | 30-day FTR | 1- Patient characteristics: demographic characteristics and comorbidity.  2-Hospital characteristics: hospital medicare case-mix, teaching status, location, bed size, ownership, total discharge, area wage index, managed care penetration, percentage of patient likely to be associated with complication (gender, race, insurance, age group)  3-Staffing: nurse aid hours per patient day, clerical staff per patient day and administrative staff per patient day | Hospital | The results from the pooled cross-sectional analysis shows that RN hours per patient day were negatively and significantly associated with FTR. Across all hospital each additional RN hour per patient day is associated with a FTR reduction of 0.47 percentage point and each additional RN + LVN hours per patient day is associated with a FTR reduction of 0.39 percentage point (*p* < 0.05).  In the linear spline model, the association between nurse staffing and FTR was not significant. As nurse staffing rose, its effect on FTR decrease. |
|  | Twigg, Duffield, Bremner, Rapley and Finn | 2011 (2000-2004) | Australia | Interrupted time series, multisite | Western Australia Human Resource Data Warehouse and patient discharge abstract | Patients who underwent surgical procedure from 3 hospitals | Nurse staffing: RN hours per patient day | Shock and cardiac arrest  In-hospital mortality  In-hospital FTR | 1-Hospitalization characteristics: season, time period and square of time periode, time periode/hospital (or ward) and time periode squared/ hospital (or ward) interactions  2-Hospital characteristics: hospital of admission (1, 2 or 3) or ward of admission (A, B, C or D) | Hospital and ward | There was no association between RN hours per patient day and shock and cardiac arrest (risk ratio 1.05 95%IC 0.64 – 1.71) and FTR (risk ratio 1.08 95%IC 0.78 – 1.52) at hospital and ward levels.  There was no association between each hospital RN hours per patient day and in-hospital mortality at ward and by hospital, but the association was significant when hospital are aggregated (risk ratio 0.75 95%IC 0.59 – 0.96). |
|  | Van den Heede, Sermeus, Diya, Clarke, Lesaffre, Vleudels and Aiken | 2009 (2003) | Belgium | Cross-sectional, multisite | Belgian Nursing Minimum Data set and Belgian Hospital Discharge Dataset | 260,923 patients who underwent general, orthopedic or vascular surgery from 115 hospitals | Nurse staffing: acuity adjusted RN hours per patient day.  Nurse education: proportion of RN with a bachelor’s degree or higher. | In-hospital mortality  In-hospital FTR  Shock or cardiac arrest | 1-Patient characteristics: age, sex, surgery type, comorbidities.  2-Hospital characteristics: teaching status (for nurse education only), technology level and hospital size. | Hospital | Nether RN hours per patient day and proportion of nurse with a bachelor’s degree were associated to shock and cardiac arrest (OR 1.43 95%IC 0.93 – 2.21), in-hospital FTR (OR 1.07 95%IC 0.80 – 1.44) and in-hospital mortality (OR 0.99 95%IC 0.45 – 2.14). |
|  | Van den Heede, Lesaffre, Diya, Vleugels, Clarke, Aiken and Sermus | 2009 (2003) | Belgium | Cross-sectional, multisite | Belgian Nursing Minimum Data set and Belgian Hospital Discharge Dataset | 9,054 patient who underwent elective cardiac surgery from 28 cardiac centers. | Nurse staffing: RN hours per patient day adjusted for nursing care intensity (in ICU and general postoperative ward separately).  Nurse education: proportion of RN with a bachelor’s degree or higher in ICU and general postoperative ward separately). | In-hospital mortality | 1-Patient characteristics: age, sex, type of procedure and risk of mortality  2- Hospital characteristics: cardiac procedural volume  3-Intensity of nursing care: 23 B-NMDS items | Ward | Nurse staffing in postoperative ICU had no significant impact on in-hospital mortality.  A greater number of RN HPPDs in postoperative general nursing units (*p*<0.0001) and proportion of RNs with a Bachelor’s degree on general units (*p*=0.0012) were associated with lower in-hospital mortality. |
|  | Wiltse Nicey, Sloane and Aiken | 2013 (2005-2007) | USA | Cross-sectional, multisite | Nurse survey, patient discharge abstract and AHA Annual Survey | 20,409 patients who underwent abdominal aortic surgery from 517 hospitals | Hospital characteristics: Hospital surgical volume  Nurse staffing: mean RN-to-patient ratio on the last shift  Nurse education: proportion of RN holding a bachelor’s degree or higher  Patient care environment based on the practice environment scales of the Nursing Work Index (PES-NWI) | 30-day mortality  30-day FTR | 1- Patient characteristics: age, sex, race, transfer status, type of medical insurance, surgery type (open vs endovascular), type of admission and comorbidities.  2- Hospital characteristics: teaching status and technology level. | Hospital | In the fully adjusted model, patient care environment (OR0.89 95%IC 0.800 – 0.989) and hospital volume (OR 0.09 95%IC 0.036-0.233) are significantly associated to mortality. Patient care environment (OR 0.90 95%IC 0.804 – 0.998) and hospital volume (OR 0.51 95%IC 0.390 – 0672) were also associated to FTR.  In the fully adjusted model, nurse staffing and nurse education were not associated to either of the complications.  A significant interaction between nurse staffing and hospital volume was found. In low-volume hospitals the effect of staffing is virtually nil, whereas in high-volume hospitals adding one additional patient per nurse increases the odds of patient death by a factor of 1.13 and the odds of FTR by a factor of 1.10. |
|  | Yasunaga, Hashimoto, Horiguchi, Miyata and Matsuda | 2012 (2007-2008) | Japan | Cross-sectional, multisite | Survey of Medical Institutions data and Diagnosis Procedure Combination (DPC) database | 131,394 patients who underwent elective lung lobectomy, esophagectomy, gastrectomy, colorectal surgery, hepatectomy or pancreatectomy for cancer from 855 healthcare center | Nurse staffing: categorical variable of physician and nurse ratio (group A: below median physician bed ratio and below nurse bed ratio, group B: below median physician bed ratio and above median nurse bed ratio, group C: above median physician bed ratio and below nurse bed ratio, and group D above median physician bed ratio and above nurse bed ratio | In-hospital mortality  In-hospital FTR | 1-Patient characteristics: age, sex and comorbidities  Hospital characteristics: volume | Hospital | The results of logistic regression analysis for FTR shows that after adjustment, group A and group D (OR 0.76 95%IC 0.63 – 0.90) were significantly different but not between group A and B or group A and C.  In a similar analysis on in-hospital mortality, group D showed a significantly lower mortality compared to group A (OR 0.82 95%IC 0.71 – 0.95) but not between group A and B or group A and C. |

TR, Failure to rescue; FTE, full-time equivalent; LPN, license practical nurse; LVN, license vocational nurse; RN, registered nurse; USA, United Stated of America
