# Supplementary material for: Are hospital nurse staffing practices associated with postoperative cardiac events and death? A systematic review
Source: PLoS One. 2019 Oct 17;14(10):e0223979. doi: 10.1371/journal.pone.0223979 (PMC6797123; doi:10.1371/journal.pone.0223979)
Supplement: S3 Table — (DOCX) [file pone.0223979.s003.docx]

| **S3. Methodological quality assessment** | | | | | | | | | | | | |
| --- | --- | --- | --- | --- | --- | --- | --- | --- | --- | --- | --- | --- |
| **Authors** | **CR1** | **CR2** | **CR3** | **CR4** | **CR5** | **CR6** | **CR7** | **CR8** | **CR9** | **CR10** | **CR11** | **Total** |
| Aiken & al. (2002)^1^ [51] | **1** | **1** | **1** | **1** | **1** | **1** | **1** | **1** | **n/a** | **n/a** | **n/a** | **8** |
| Aiken & al. (2003)^1^ [52] | **1** | **1** | **1** | **1** | **1** | **1** | **1** | **1** | **n/a** | **n/a** | **n/a** | **8** |
| Aiken & al. (2008)^1^ [53] | **1** | **1** | **1** | **1** | **1** | **1** | **1** | **1** | **n/a** | **n/a** | **n/a** | **8** |
| Aiken & al. (2011)^1^ [54] | **1** | **1** | **1** | **1** | **1** | **1** | **1** | **1** | **n/a** | **n/a** | **n/a** | **8** |
| Aiken & al. (2014)^1^ [55] | **1** | **1** | **1** | **1** | **1** | **1** | **1** | **1** | **n/a** | **n/a** | **n/a** | **8** |
| Aiken & al. (2017)^1^ [65] | **1** | **1** | **1** | **1** | **1** | **1** | **1** | **1** | **n/a** | **n/a** | **n/a** | **8** |
| Ball & al. (2018)^1^ [56] | **1** | **1** | **1** | **1** | **1** | **1** | **1** | **1** | **n/a** | **n/a** | **n/a** | **8** |
| Berney & Needleman (2006)^1^ [24] | **1** | **0** | **1** | **1** | **1** | **1** | **1** | **1** | **n/a** | **n/a** | **n/a** | **7** |
| Carthon & al. (2012)^1^ [57] | **1** | **1** | **0** | **1** | **1** | **1** | **1** | **1** | **n/a** | **n/a** | **n/a** | **7** |
| Cho & al. (2015)^1^ [20] | **1** | **1** | **1** | **1** | **1** | **1** | **1** | **1** | **n/a** | **n/a** | **n/a** | **8** |
| Dang & al. (2002)^1^ [40] | **1** | **1** | **0** | **1** | **1** | **1** | **1** | **1** | **n/a** | **n/a** | **n/a** | **7** |
| Dimick & al. (2001)^1^ [39] | **1** | **1** | **1** | **1** | **1** | **1** | **1** | **1** | **n/a** | **n/a** | **n/a** | **8** |
| Diya & al. (2010)^1^ [46] | **1** | **1** | **1** | **1** | **1** | **1** | **1** | **1** | **n/a** | **n/a** | **n/a** | **8** |
| Diya & al. (2012)^1^ [47] | **1** | **1** | **1** | **1** | **1** | **1** | **1** | **1** | **n/a** | **n/a** | **n/a** | **8** |
| Elkassabany & al. (2016)^1^ [61] | **1** | **1** | **0** | **1** | **1** | **U** | **1** | **1** | **n/a** | **n/a** | **n/a** | **6** |
| Friese & al. (2008)^1^ [58] | **1** | **1** | **1** | **1** | **1** | **1** | **1** | **1** | **n/a** | **n/a** | **n/a** | **8** |
| Ghaferi & al. (2010)^1^ [21] | **1** | **1** | **1** | **1** | **1** | **1** | **1** | **1** | **n/a** | **n/a** | **n/a** | **8** |
| Griffiths & al. (2013)^1^ [22] | **1** | **U** | **U** | **0** | **1** | **1** | **1** | **1** | **n/a** | **n/a** | **n/a** | **5** |
| Halm & al. (2005)^1^ [62] | **1** | **1** | **1** | **1** | **1** | **1** | **1** | **1** | **n/a** | **n/a** | **n/a** | **8** |
| Harless & Mark (2010)^1^ [69] | **1** | **0** | **1** | **0** | **1** | **1** | **1** | **1** | **n/a** | **n/a** | **n/a** | **6** |
| Hickey & al. (2010)^1^ [43] | **1** | **1** | **1** | **1** | **1** | **1** | **1** | **1** | **n/a** | **n/a** | **n/a** | **8** |
| Hickey & al. (2011)^1^ [44] | **1** | **0** | **1** | **1** | **1** | **1** | **1** | **1** | **n/a** | **n/a** | **n/a** | **7** |
| Kendall-Gallagher & al. (2011)^1^ [67] | **1** | **1** | **1** | **1** | **1** | **1** | **1** | **1** | **n/a** | **n/a** | **n/a** | **8** |
| Kiekkas & al. (2008)^2^ [45] | **1** | **1** | **1** | **1** | **1** | **1** | **1** | **1** | **1** | **n/a** | **1** | **10** |
| Kutney-Lee & Aiken (2008)^1^ [59] | **1** | **1** | **1** | **1** | **1** | **1** | **1** | **1** | **n/a** | **n/a** | **n/a** | **8** |
| Lane-Fall & al. (2017)^1^ [68] | **1** | **1** | **1** | **1** | **1** | **1** | **1** | **1** | **n/a** | **n/a** | **n/a** | **8** |
| Li & al. (2016)^3^ [48] | **1** | **1** | **1** | **1** | **1** | **1** | **1** | **U** | **1** | **1** | **n/a** | **9** |
| Mark & al. (2007) ^1^ [38] | **1** | **U** | **1** | **0** | **1** | **1** | **1** | **1** | **n/a** | **n/a** | **n/a** | **6** |
| Mark & Harless (2009)^1^ [23] | **1** | **0** | **U** | **1** | **1** | **1** | **1** | **1** | **n/a** | **n/a** | **n/a** | **6** |
| McCloskey & Diers (2005)^2^ [35] | **1** | **1** | **U** | **0** | **0** | **1** | **1** | **1** | **1** | **n/a** | **1** | **7** |
| Needleman & al. (2002)^1^ [30] | **1** | **1** | **1** | **1** | **1** | **1** | **1** | **1** | **n/a** | **n/a** | **n/a** | **8** |
| Neff & al. (2013)^1^ [60] | **1** | **1** | **U** | **1** | **1** | **1** | **1** | **1** | **n/a** | **n/a** | **n/a** | **7** |
| Newhouse & al. (2005)^1^ [26] | **1** | **1** | **1** | **1** | **1** | **1** | **1** | **1** | **n/a** | **n/a** | **n/a** | **8** |
| Olds & al. (2017)^1^ [50] | **1** | **1** | **1** | **1** | **1** | **1** | **1** | **1** | **n/a** | **n/a** | **n/a** | **8** |
| Ozdemir & al. (2016)^1^ [63] | **1** | **1** | **1** | **1** | **1** | **1** | **1** | **1** | **n/a** | **n/a** | **n/a** | **8** |
| Rafferty & al. (2007)^1^ [41] | **1** | **1** | **1** | **1** | **1** | **1** | **1** | **1** | **n/a** | **n/a** | **n/a** | **8** |
| Rao & al. (2017)^1^ [66] | **1** | **1** | **1** | **1** | **1** | **1** | **1** | **1** | **n/a** | **n/a** | **n/a** | **8** |
| Schreuders & al. (2015)^2^ [19] | **1** | **1** | **1** | **1** | **1** | **1** | **1** | **1** | **1** | **n/a** | **1** | **10** |
| Sochalski & al. (2008)^1^ [70] | **1** | **1** | **1** | **1** | **1** | **1** | **1** | **U** | **n/a** | **n/a** | **n/a** | **7** |
| Twigg & al. (2011)^4^ [36] | **1** | **1** | **1** | **0** | **1** | **1** | **1** | **1** | **1** | **n/a** | **n/a** | **8** |
| Van den Heede & al (2009a)^1^ [37] | **1** | **1** | **1** | **1** | **1** | **1** | **1** | **1** | **n/a** | **n/a** | **n/a** | **8** |
| Van den Heede & al (2009b)^1^ [49] | **1** | **1** | **1** | **1** | **1** | **1** | **1** | **1** | **n/a** | **n/a** | **n/a** | **8** |
| Wiltse Nicey & al. (2013)^1^ [64] | **1** | **1** | **1** | **1** | **1** | **1** | **1** | **1** | **n/a** | **n/a** | **n/a** | **8** |
| Yasunaga & al. (2012)^1^ [42] | **1** | **1** | **U** | **1** | **1** | **1** | **1** | **1** | **n/a** | **n/a** | **n/a** | **7** |
| Abbreviations:  CR: criteria, n/a: not applicable, U: uncertain, 1: yes, 0: no  Notes:  ^1^ Checklist for Analytical Cross-Sectional Studies  ^2^ Checklist for Cohort Studies  ^3^ Checklist for Case-Control Studies  ^4^ Checklist for Quasi-Experimental Studies (non-randomized experimental studies) | | | | | | | | | | | | |
